# Inferring and evaluating network medicine-based disease modules with nextflow

**DOI:** 10.1093/bioinformatics/btag223

**Published:** 2026-07-07

**Authors:** Johannes Kersting, Chloé Bucheron, Lisa M Spindler, Joaquim Aguirre-Plans, Quirin Manz, Tanja Pock, Mo Tan, Fernando M Delgado-Chaves, Cristian Nogales, Harald H H W Schmidt, Jörg Menche, Andreas Maier, Jan Baumbach, Emre Guney, Markus List

**Affiliations:** Data Science in Systems Biology, TUM School of Life Sciences, Technical University of Munich, Freising, Germany; Max Perutz Labs, Department of Structural and Computational Biology, University of Vienna, Vienna, Austria; Vienna Biocenter PhD Program, a Doctoral School of the University of Vienna and the Medical University of Vienna, Vienna, Austria; Ludwig Boltzmann Institute for Network Medicine, University of Vienna, Vienna, Austria; Data Science in Systems Biology, TUM School of Life Sciences, Technical University of Munich, Freising, Germany; Discovery and Data Science (DDS) Unit, STALICLA SL, Barcelona, Spain; Data Science in Systems Biology, TUM School of Life Sciences, Technical University of Munich, Freising, Germany; Data Science in Systems Biology, TUM School of Life Sciences, Technical University of Munich, Freising, Germany; Data Science in Systems Biology, TUM School of Life Sciences, Technical University of Munich, Freising, Germany; Institute for Computational Systems Biomedicine, University of Hamburg, Hamburg, Germany; Max Perutz Labs, Department of Structural and Computational Biology, University of Vienna, Vienna, Austria; Ludwig Boltzmann Institute for Network Medicine, University of Vienna, Vienna, Austria; Department of Pharmacology and Personalised Medicine, Maastricht University, Maastricht, The Netherlands; Max Perutz Labs, Department of Structural and Computational Biology, University of Vienna, Vienna, Austria; Ludwig Boltzmann Institute for Network Medicine, University of Vienna, Vienna, Austria; Faculty of Mathematics, University of Vienna, Vienna, Austria; Research Center for Molecular Medicine of the Austrian Academy of Sciences, Vienna, Austria; Institute for Computational Systems Biomedicine, University of Hamburg, Hamburg, Germany; Institute for Computational Systems Biomedicine, University of Hamburg, Hamburg, Germany; Discovery and Data Science (DDS) Unit, STALICLA SL, Barcelona, Spain; Data Science in Systems Biology, TUM School of Life Sciences, Technical University of Munich, Freising, Germany; Munich Data Science Institute (MDSI), Technical University of Munich, Garching, Germany

## Abstract

**Motivation:**

Most diseases result from complex molecular interactions of genes and proteins. Various network-based methods characterize these mechanisms by expanding seed genes into disease modules. Their underlying algorithmic strategies differ, making it difficult to determine which of the created modules are most useful or biologically plausible.

**Results:**

To address this challenge, we developed an all-in-one pipeline that handles installation, input preparation, execution, and systematic evaluation of six widely used module detection tools, considering module topology, functional coherence, robustness, and the capacity to recover seeds. To showcase the value of our pipeline and provide guidance to potential users, we conducted a comprehensive evaluation across 50 different disease-network combinations, revealing substantial variability among the derived disease modules, driven by both network and algorithm choices. We show that methods are robust to minor perturbations but struggle to recover omitted seeds. None consistently outperforms all others, underscoring the need for careful method selection. Our work enables the systematic comparison of disease module discovery approaches and promotes reproducible network medicine research. Integrated into the nf-core project, it is intended as an extendable, long-term resource for tracking progress in the field.

**Availability and Implementation:**

The pipeline is implemented in Nextflow. Code and documentation are available through GitHub (https://github.com/nf-core/diseasemodulediscovery) and the nf-core website (https://nf-co.re/diseasemodulediscovery). Code and data used for demonstrating the pipeline are available through GitHub (https://github.com/REPO4EU/modulediscovery_demonstration).

## 1 Introduction

Diseases often emerge from complex disturbances in the interactions between different biomolecules. These molecular interactions collectively form the human interactome, which can be represented as a network in which nodes correspond to proteins, DNA, or metabolites, and edges model the interactions between them. Studying the interactome, most frequently focusing on protein-protein interactions (PPIs), and its disease-related perturbations is a central area in network medicine. A key insight of the field is that disease-associated genes, or more precisely, their protein products, are not randomly scattered throughout the interactome. Instead, they tend to cluster within local neighborhoods, forming so-called disease modules (Ghiassian *et al*. 2015). Disease modules can be used to characterize diseases by their molecular mechanisms, rather than traditional organ- or symptom-based definitions. Combined with network pharmacology they can support discovering novel drug targets and drug candidates that address the underlying pathological processes, which may be further enhanced through synergistic drug effects (Nogales *et al*. 2022).

**Figure 1:**
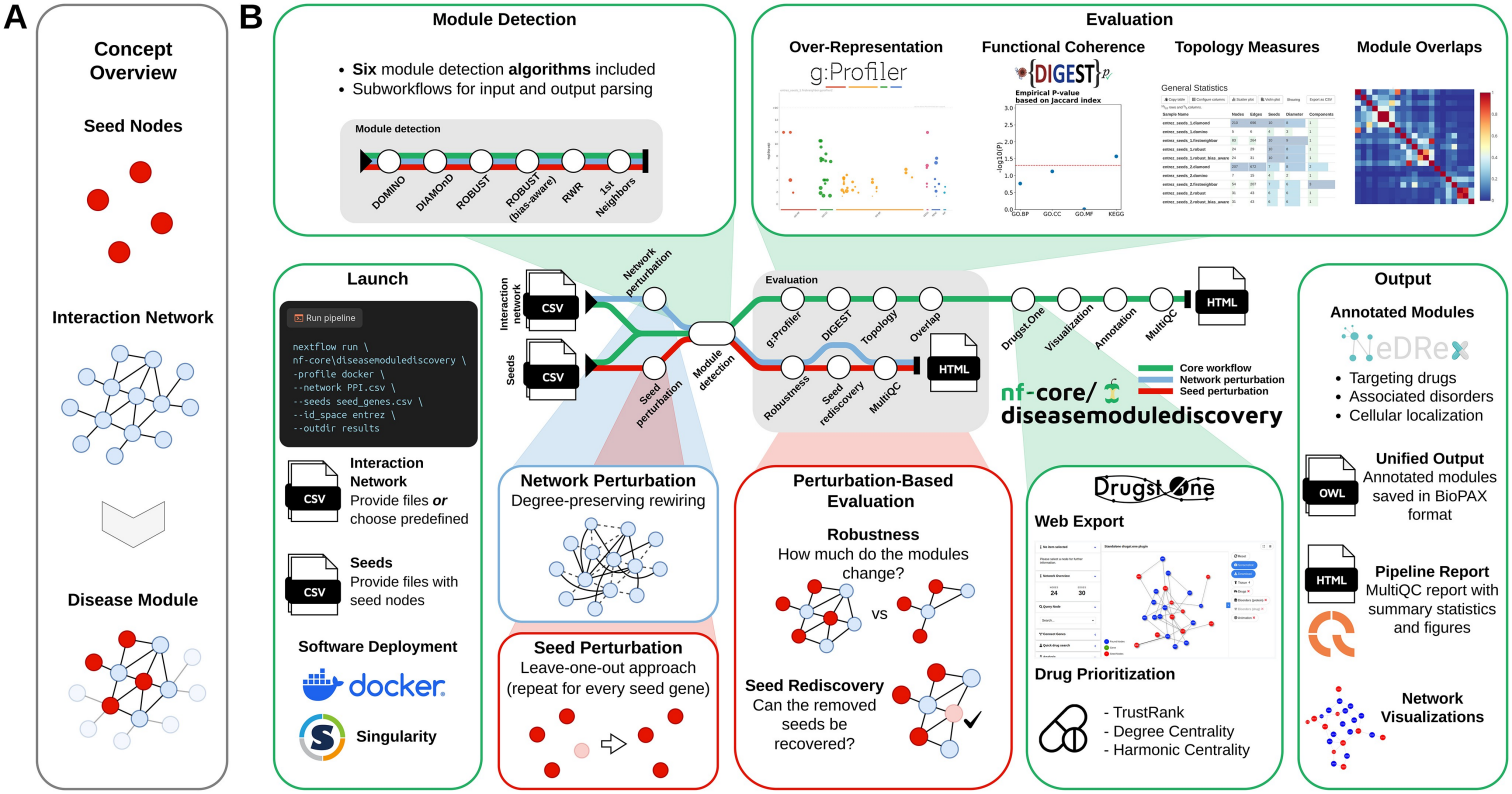
(A) Inferring disease modules based on a set of seed nodes and an interaction network. (B) Our pipeline accepts multiple seed files and networks as input. Seed files must be supplied by the user; networks can either be supplied or selected from a list of available options. The pipeline runs six widely used AMIMs based on the provided input, including DOMINO, DIAMOnD, ROBUST and its bias-aware implementation, a 1st Neighbors approach, and random walk with restart (RWR). This results in one module for every combination of seed file, network file, and AMIM specified. The resulting modules are evaluated through over-representation (g: Profiler) and functional coherence (DIGEST) analyses, network topology measures, and pairwise node set overlaps. Degree-preserving network rewiring is applied to assess potential node-degree biases. A leave-one-out analysis on the seed set evaluates the robustness of the AMIMs to small input perturbations and calculates a rediscovery rate. Drug candidates are prioritized using various network-based algorithms by interfacing with Drugst.One. The pipeline also generates network visualizations of the modules, annotates them with supplementary biological information (queried from NeDRex), saves them in a range of standardized output formats, and provides an HTML summary report of the entire run using MultiQC.

Numerous algorithmic approaches for disease module identification, also referred to as active module identification methods (AMIMs), have been proposed. They typically start with a set of known disease-associated genes or proteins (referred to as seeds) and expand them into disease modules by incorporating additional nodes from the interactome (see Fig. 1A). However, choosing the right method is not trivial, as they frequently follow different rationales on how a disease module is best identified. A 1st Neighbors approach simply expands the seed set by adding nodes that directly interact with at least one seed node. DIAMOnD (Ghiassian *et al*. 2015) expands the seed nodes by repeatedly adding the node with the most significant number of interactions with the current seeds. DOMINO (Levi *et al*. 2021) first partitions the interactome into non-overlapping clusters and further refines those with a higher-than-expected number of seeds. ROBUST (Bernett *et al*. 2022) repeatedly connects the seed nodes into a single connected component. Nodes that appear in a sufficiently large number of solutions are incorporated into the final module. Its research bias-aware implementation (Sarkar *et al*. 2023) works similarly, but penalizes the inclusion of overstudied proteins. Network propagation methods, such as random walk with restart (RWR) (Köhler *et al*. 2008), simultaneously expand the seed set in all directions, e.g., until they constitute a single connected component.

This method diversity is required, as the best approach may depend on both the topological shape of the disease mechanism (e.g., organized around a single hub, describing a pathway, or more complex patterns) but also on the abundance of and confidence in known seed genes. For example, if a disease mechanism is characterized by multiple disjoint subnetworks, a method that is based on connecting seeds is inappropriate. If only a few disease-associated genes are known, a more explorative method may yield better results than a conservative one. For most disease mechanisms, we can only speculate on these properties, necessitating a framework that allows assessing multiple approaches and comparing them effectively.

Applying and comparing several AMIMs is hindered by method-specific installation processes, input requirements, and output formats, making manual execution cumbersome. Moreover, input perturbation-based evaluations are computationally demanding and necessitate efficient parallelization to remain practical. Furthermore, although AMIMs have been benchmarked in multiple studies (Choobdar *et al*. 2019, Lazareva *et al*. 2021, Yang *et al*. 2024), these evaluations are based on predefined benchmarking datasets and thus do not permit assessment of the methods within a specific user context.

As part of the Horizon Europe project REPO4EU (https://repo4.eu), we sought a robust workflow that includes disease module identification, systematic evaluation, visualization, and drug candidate prioritization. To this end, we present a novel Nextflow pipeline that autonomously executes all these steps, parallelizes tasks where possible, and manages dependencies via containers, simplifying the setup and ensuring reproducibility. It is released as an independent pipeline within nf-core (Ewels *et al*. 2020), a collection of community-curated bioinformatics pipelines built with Nextflow. To showcase the analytical power of our pipeline, we applied it to a combination of ten diverse input networks and five seed sets. In addition to demonstrating the volume and diversity of results that can be generated from a single pipeline run, it also enabled us to make several key observations: (i) reflecting their distinct modeling approaches, different AMIMs produce disease modules with markedly different topologies; (ii) most AMIMs produce disease modules that are specific to their corresponding diseases, although this specificity largely arises from the seed set; (iii) the choice of input network has a major influence on the resulting modules; (iv) most AMIMs exhibit robustness to small input perturbations; but (v) generally struggle to reliably rediscover omitted seeds; (vi) no AMIM consistently outperformed the others across all categories, underscoring the need for careful method selection.

## 2 Results

Figure 1B provides a detailed overview of our pipeline. To showcase its functionality and highlight characteristics of the integrated AMIMs, we applied it to five different seed sets combined with ten PPI networks, yielding 50 input combinations. The seed sets consisted of disease-associated genes for Huntington’s disease (HD), ulcerative colitis (UC), Crohn’s disease (CD), amyotrophic lateral sclerosis (ALS), and lung adenocarcinoma (LUAD), obtained from DisGeNET (Piñero *et al*. 2020) (see Figure S1B, Table S2, and Methods, available as supplementary data at *Bioinformatics* online). The selection of diseases was inspired by previous benchmarking approaches (Lazareva *et al*. 2021, Bernett *et al*. 2022, Sarkar *et al*. 2023). As interactome networks, we used human PPIs from STRING (Szklarczyk *et al*. 2023), BioGRID (Oughtred *et al*. 2021), HIPPIE (Alanis-Lobato *et al*. 2017), IID (Kotlyar *et al*. 2022), and NeDRex (Sadegh *et al*. 2021) in different subset configurations. These networks are directly available through the pipeline interface (see Figure S1A, Table S1, and Supplementary Methods, available as supplementary data at *Bioinformatics* online).

### 2.1 AMIMs produce modules with distinct topologies

As expected, the AMIMs in the pipeline generated disease modules with distinct topological characteristics.

**Figure 2:**
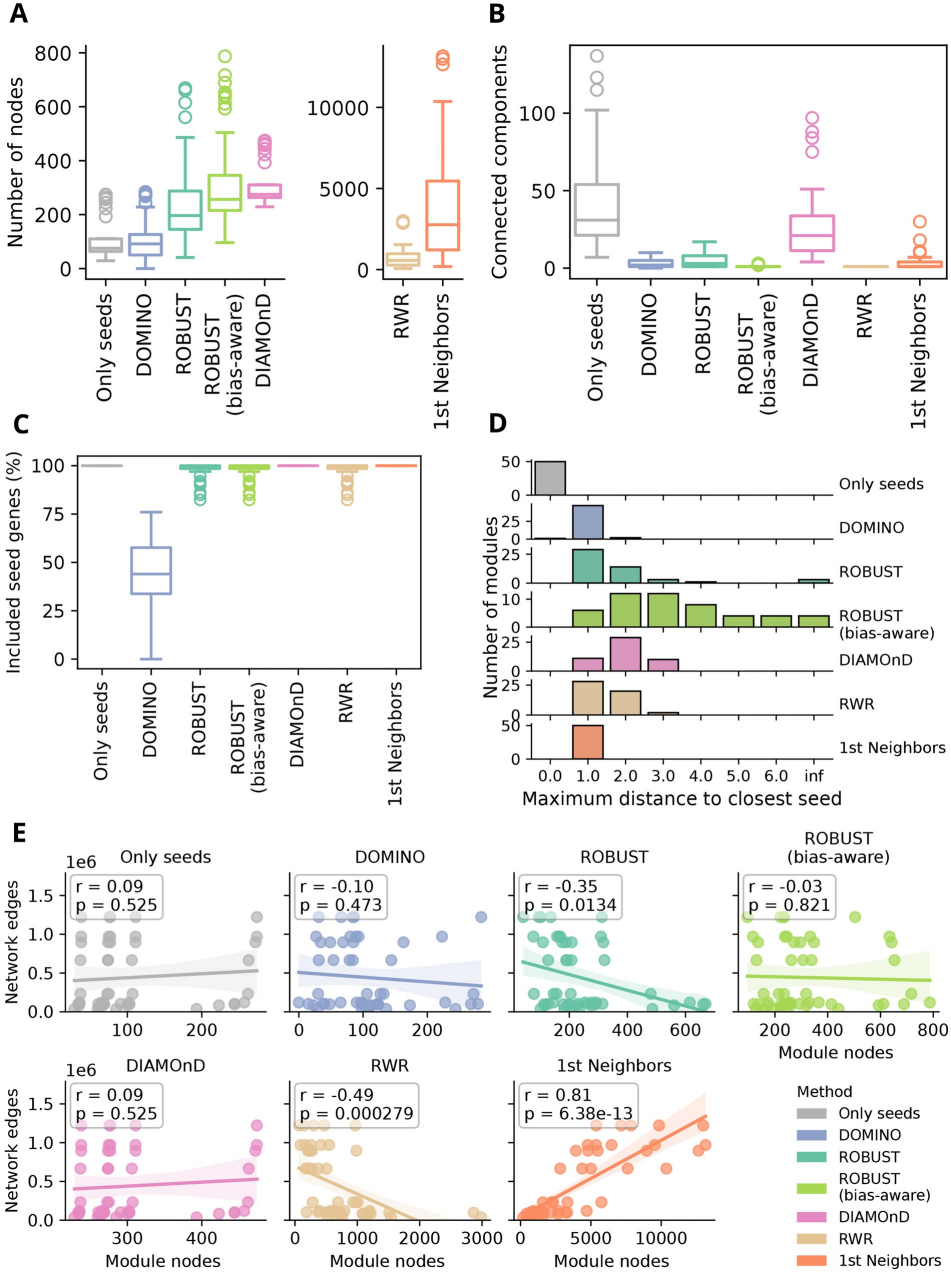
Topological properties of disease modules inferred using different methods. All measures are based on the subnetwork induced by the disease module nodes. Subfigures A-D provide summaries across all 50 input combinations used. (A) Disease module size, defined as the number of nodes included in the module. (B) Number of connected components within each disease module. (C) Proportion of seed nodes retained in the final disease module. (D) Maximum distance from any added (non-seed) node to its nearest seed node, serving as a measure of explorativeness. A distance of 0 indicates that no additional nodes were included; “inf” (infinite) refers to nodes completely isolated from the seed set. (E) Correlation between disease module size and the number of edges in the input network. *r* indicates the Pearson correlation and *p* the corresponding p-value. Each point represents a single disease module.

DOMINO produces the smallest disease modules, followed by ROBUST, ROBUST (bias-aware), and DIAMOnD (Fig. 2A). RWR and 1st Neighbors can yield considerably larger modules, which can include thousands, and in the case of 1st Neighbors, even over ten thousand nodes. Naturally, module size is influenced by the input data: all methods tend to produce larger modules when provided with larger seed sets (Figure S2, available as supplementary data at *Bioinformatics* online). The effect of input network size varies by method (Fig. 2E). In larger networks, seed nodes have more interactors, which leads to larger modules for the 1st Neighbors approach. Conversely, methods designed to connect seed nodes, such as ROBUST and RWR, generate smaller modules, as additional nodes and edges allow for more efficient paths between seeds. The size of the modules produced by DOMINO, DIAMOnD, and ROBUST (bias-aware) is not significantly influenced by network size. This is notable in the case of ROBUST (bias-aware), since it aims to connect the seeds, which should be easier in larger networks. A likely explanation is that ROBUST (bias-aware) penalizes the inclusion of highly connected, overstudied hub genes, thereby avoiding shortcuts in larger networks (Blumenthal *et al*. 2024).

Interestingly, the seed nodes themselves never form a single connected component (Fig. 2B). This may be due to the incompleteness of the interactome, the involvement of multiple independent mechanisms, or the existence of nodes relevant to the disease mechanism that are not yet included in the seed set, motivating the use of disease module inference algorithms to complete them (Buzzao *et al*. 2022). The modules produced by DIAMOnD often consist of many components, but fewer than the seeds form alone. DOMINO, ROBUST, and 1st Neighbors can yield multiple connected components, but generally return fewer than DIAMOnD. ROBUST (bias-aware) almost always returns a single connected component, whereas RWR does so by design (termination criterion of the pipeline implementation; see Supplementary Methods, available as supplementary data at *Bioinformatics* online).

Except for DIAMOnD and 1st Neighbors, the evaluated AMIMs do not necessarily retain all seed nodes in their modules (Fig. 2C). ROBUST, ROBUST (bias-aware), and RWR exclude seeds only if not part of the largest connected component (LCC) of the input network. In contrast, DOMINO frequently omits seeds, with a median retention rate of less than 50%.

Figure 2D illustrates the explorativeness of the AMIMs, described by the frequency with which they add nodes distant from the seeds. As 1st Neighbors only includes direct interactors, this distance is always one. Notably, DOMINO tends to incorporate primarily direct interactors, although no such restriction is built into the method. RWR and ROBUST modules are primarily composed of direct interactors, but second neighbors are also commonly included. DIAMOnD typically incorporates at least second neighbors and occasionally even third neighbors. ROBUST (bias-aware) is by far the most explorative method, including nodes up to six edges away from any seed. This behavior is again likely explained by the avoidance of shortcuts through overstudied hub genes.

The observation that the modules generated by different AMIMs exhibit distinct topologies is consistent with observations from previous studies (Choobdar *et al*. 2019) and underscores the value of applying and comparing multiple methods for a single use case, as their solutions may be complementary. Please note that some described properties may vary depending on the specific parameterization of the AMIM (see Supplementary Note 1, available as supplementary data at *Bioinformatics* online).

### 2.2 The included seed nodes are the primary reason for disease-specific modules

Despite the pronounced size differences between the input networks (Figure S1A, available as supplementary data at *Bioinformatics* online), disease modules for the same disease should be disease-specific and, therefore, group when clustered based on their similarity.

**Figure 3:**
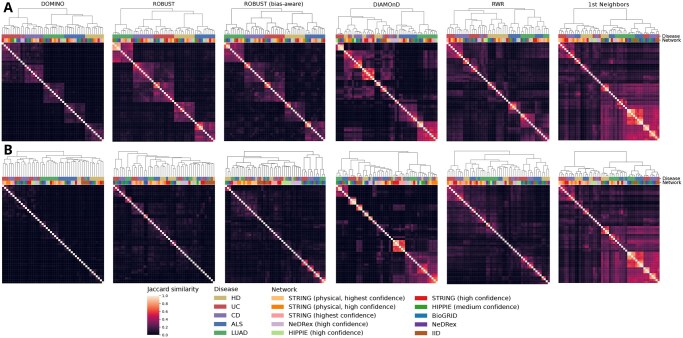
Heatmaps and hierarchical clusterings for each AMIM based on the pair-wise node set Jaccard similarities of modules inferred using different diseases and networks. (A) Considering all module nodes. (B) Only considering added nodes (no seed nodes).

Figure 3A (see Figure S3 for a combined heatmap, available as supplementary data at *Bioinformatics* online) confirms this expectation for modules generated by DOMINO, ROBUST, and ROBUST (bias-aware). Additionally, the modules for UC and CD exhibit similarities, consistent with both being subtypes of Inflammatory Bowel Disease (IBD), which share mechanistic features (Sartor, 2006). In contrast, modules produced by DIAMOnD, RWR, and 1st Neighbors fail to cluster cleanly by disease. Since these methods generate larger modules (Fig. 2A), the loss of specificity is likely due to the inclusion of excessive noise.

However, achieving disease-specific modules is a relatively low bar because the seed sets are already disease-specific and are typically retained in the resulting modules (Fig. 2C). To address this, the pipeline calculates module similarities while excluding the seed nodes and considering only the added nodes. Under this criterion (see Fig. 3B and Figure S4 for a combined heatmap, available as supplementary data at *Bioinformatics* online), the modules of most AMIMs no longer cluster by disease, indicating that the observed disease-specificity of the full modules mainly arises from the shared seed sets, rather than from the disease-specific added nodes. The only exception is DOMINO, which largely maintains disease specificity, albeit with rather low overlaps (<0.25) off the diagonal and difficulty distinguishing the IBD subtypes.

In line with these results, the median overlap between modules generated with the same AMIM and seed set but different networks is consistently below 0.5 (Figure S5, available as supplementary data at *Bioinformatics* online). This indicates that the modules are highly dependent on the chosen network. Consequently, a module inferred using one network is unlikely to be reproducible with a different network.

### 2.3 Most AMIMs are robust to leave-one-out perturbations

Because gene–disease association data inevitably contain noise and false positives, disease modules should not rely excessively on individual seeds (Ghiassian *et al*. 2015). To evaluate this, the pipeline performs a leave-one-out analysis, sequentially removing each seed node from the input and comparing the perturbed modules to the original ones.

**Figure 4:**
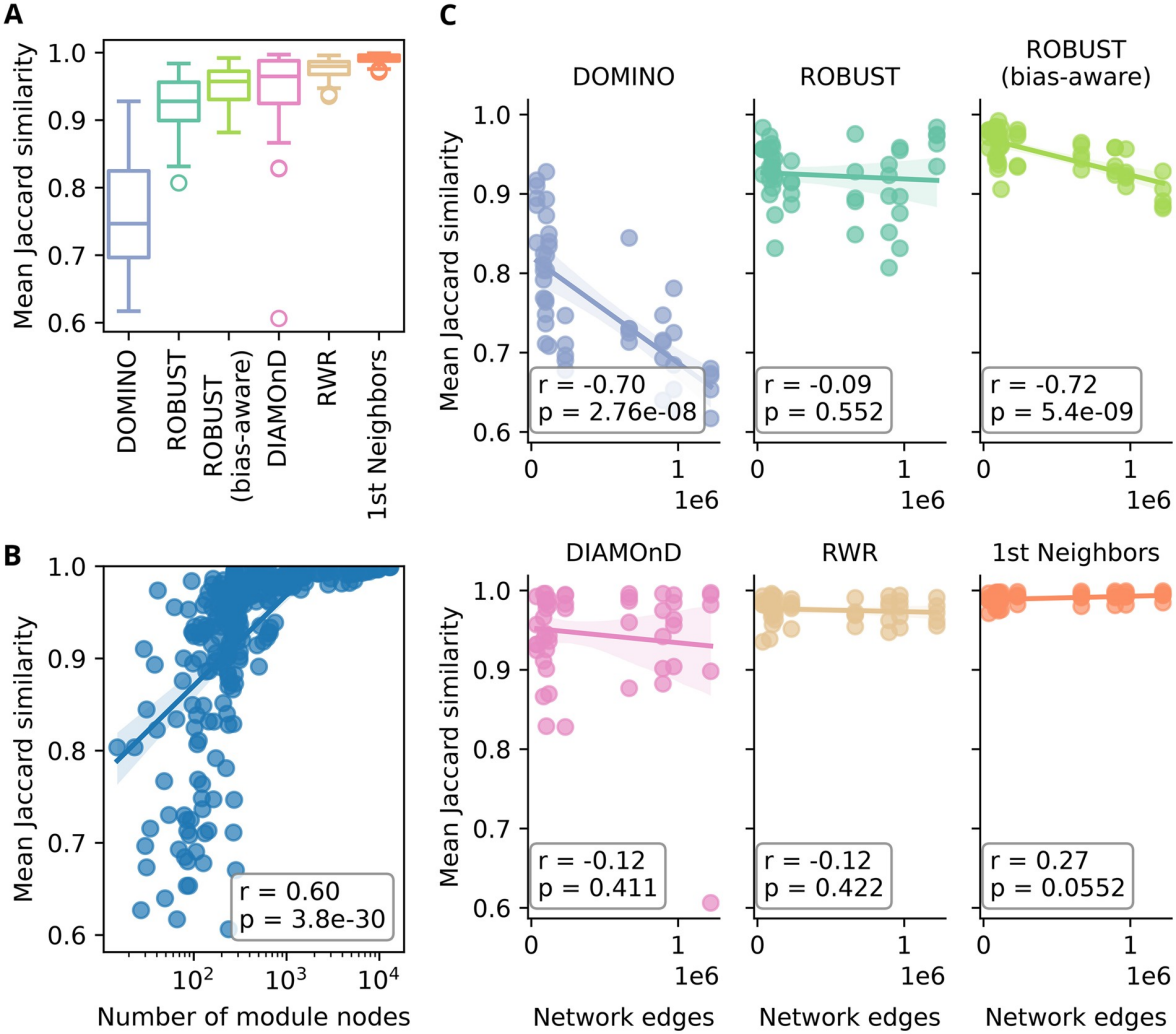
Robustness of AMIMs to leave-one-out perturbations of the input seed set. Robustness is quantified as the mean Jaccard similarity between the node set of the original module and those of its perturbed variants, with higher values indicating greater robustness. (A) Boxplots summarize results aggregated across different seed set–network combinations. (B) Correlation between robustness and module size. (C) Correlations between robustness and input network size (measured by the number of edges). *r* indicates the Pearson correlation and *p* the corresponding p-value. Each point represents a single disease module.

Figure 4A demonstrates that most AMIMs are robust to the omission of individual genes, with median mean Jaccard similarities exceeding 0.9. The only exception is DOMINO, which exhibits lower robustness (median ∼0.75). Notably, a clear positive relationship exists between module size and robustness (Fig. 4B and Figure S6, available as supplementary data at *Bioinformatics* online). Consequently, AMIMs that generate larger modules (Fig. 2A) tend to yield more robust results. Moreover, with the exception of DOMINO, AMIMs also show increased robustness when provided with larger seed sets (Figure S7, available as supplementary data at *Bioinformatics* online). In contrast, the number of edges in the input network is negatively correlated with robustness, except for 1st Neighbors (Fig. 4C). This effect is particularly pronounced for DOMINO and ROBUST (bias-aware).

### 2.4 AMIMs cannot reliably rediscover disease genes

In addition to assessing robustness to small input perturbations, the pipeline uses leave-one-out seed perturbations to calculate a rediscovery rate, defined as the fraction of removed seeds that are reincluded in the module by the AMIM (see Methods). Since the seed genes are known to be associated with the corresponding disease, this metric provides an estimate of how effectively an AMIM might discover genes with previously unknown disease associations.

**Figure 5:**
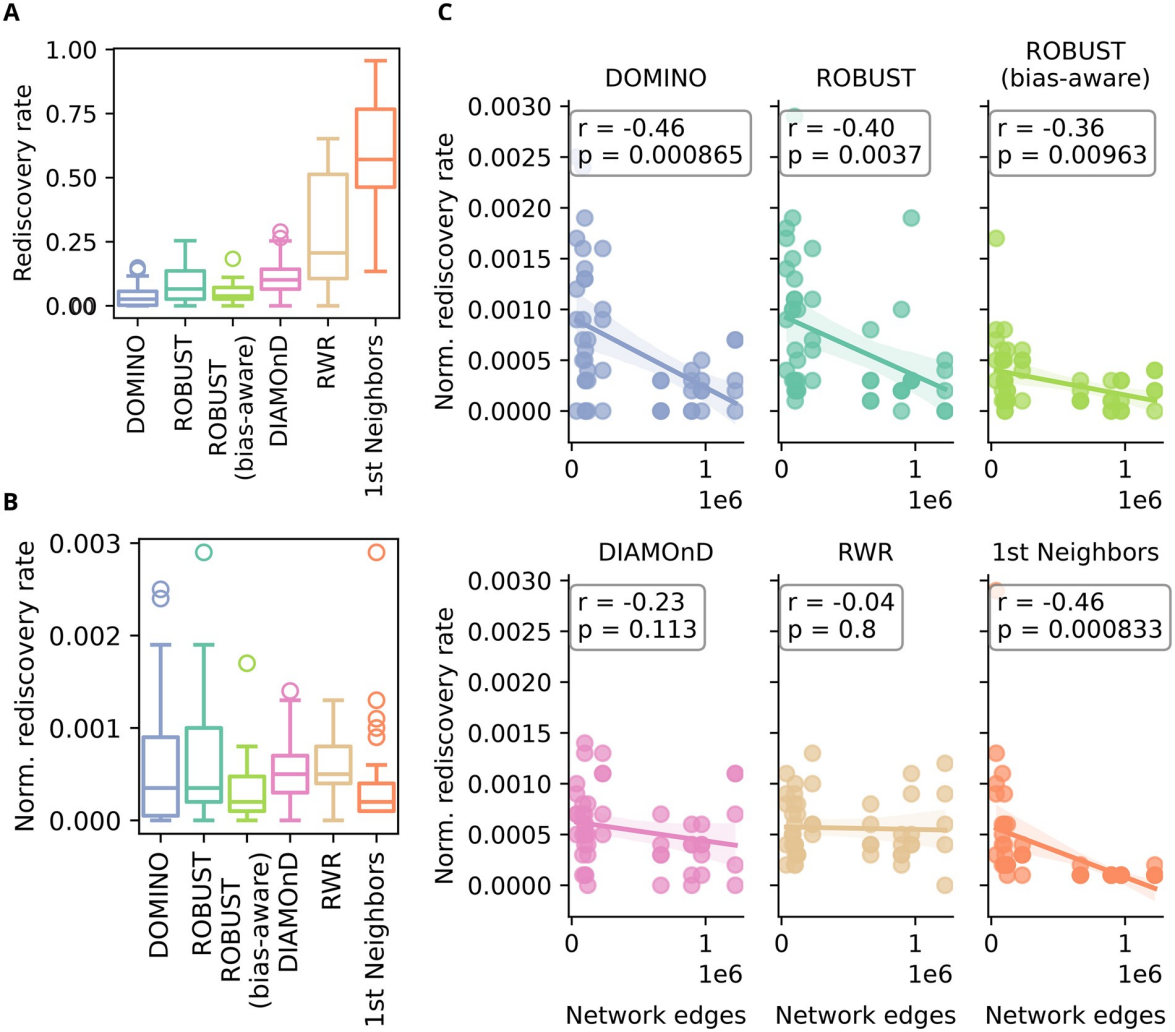
Results of the seed rediscovery analysis. (A) Distributions of seed rediscovery rates and (B) normalized seed rediscovery rates across different AMIMs. A single boxplot summarizes results aggregated across different seed set–network combinations. (C) Correlations between normalized seed rediscovery rate and input network size (measured by the number of edges). *r* indicates the Pearson correlation and *p* the corresponding p-value. Each point represents a single disease module.

As shown in Fig. 5A, the highest rediscovery rate is achieved by 1st Neighbors, which reaches a median rediscovery rate above 50%. In contrast, the median rediscovery rates of other AMIMs are substantially lower, dropping below 10% for DOMINO, ROBUST, and ROBUST (bias-aware).

However, 1st Neighbors modules tend to become very large (Fig. 2A), and the probability of re-including a specific node increases by chance when module size grows. To account for this effect, the pipeline reports a normalized rediscovery rate (Fig. 5B), obtained by dividing the rediscovery rate by the number of nodes in the module (see Methods). After normalization, differences between methods are less pronounced, with DIAMOnD and RWR achieving the highest median normalized rediscovery rates, while ROBUST (bias-aware) and 1st Neighbors perform the worst. Notably, ROBUST (bias-aware) exhibits reduced performance compared to its original implementation, likely because disease-associated genes tend to be well- or even overstudied (Schaefer *et al*. 2015), and therefore are penalized by the method.

We hypothesized that seed rediscovery might depend on the underlying disease, with some diseases being more easily recovered than others. However, this is not the case (Figure S8, available as supplementary data at *Bioinformatics* online). In contrast, network choice has a clear impact, with network size consistently showing a negative correlation with the normalized rediscovery rate (Fig. 5C).

Taken together, this demonstrates that while AMIMs have the potential to identify relevant genes, the rediscovery rates are rather low and differences between methods are mainly driven by module size.

### 2.5 Node inclusion becomes increasingly degree-driven for larger networks

Lazareva *et al*. (2021) showed that most AMIMs do not produce biologically more meaningful results on real networks than on degree-preserving randomized networks, suggesting that these methods primarily leverage node degree information rather than specific PPIs.

To detect this behavior within our pipeline, the input network is repeatedly rewired in a degree-preserving manner (see Methods). The resulting modules are compared to those derived from the original network using the same procedure as in the leave-one-out seed perturbation analysis. However, in this case, strong deviations from the original modules are desirable, as close similarity would indicate that an AMIM primarily exploits node degree shortcuts.

**Figure 6:**
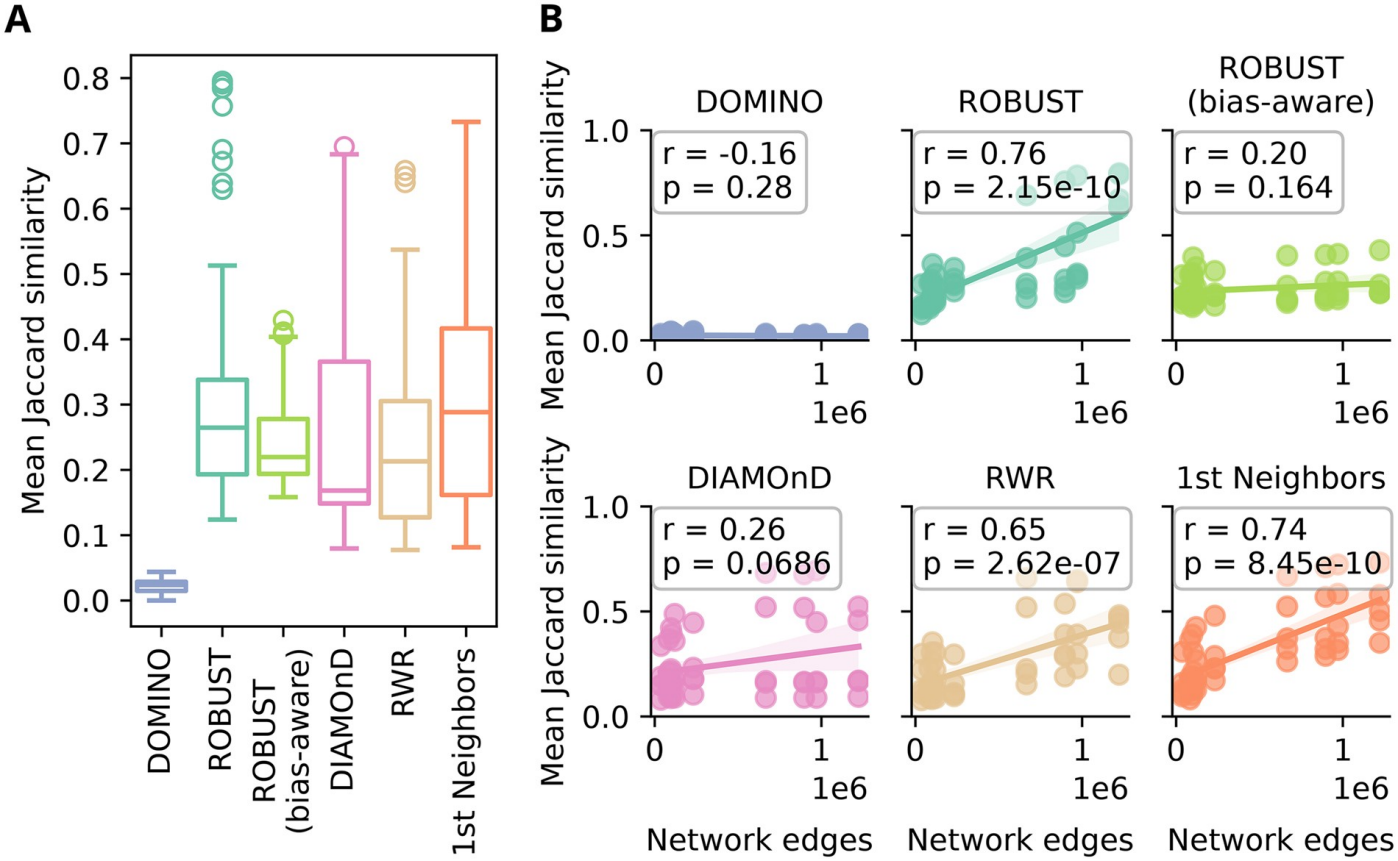
Results of the degree-preserving network rewiring analysis. High mean Jaccard similarities between the original modules and those based on the rewired networks, depicted on the *y*-axis, indicate a strong reliance on node degrees. (A) A single boxplot summarizes results aggregated across different seed set–network combinations. (B) Correlations between Jaccard similarity and input network size (measured by the number of edges). *r* indicates the Pearson correlation and *p* the corresponding *p*-value. Each point represents a single disease module.

Figure 6A summarizes the results of this analysis. Notably, AMIMs are more affected by network rewiring than by the removal of individual seed genes (median mean Jaccard similarity <0.3 compared to >0.9 for most AMIMs in the leave-one-out analysis, Fig. 4A). This indicates that none of the evaluated AMIMs rely solely on node degrees. Among the methods, DOMINO relies the least on node degrees, consistent with the findings of Lazareva **et al*.* As expected, the 1st Neighbors approach is biased by high-degree nodes, which are more likely to interact with at least one seed node by chance. Furthermore, ROBUST shows a stronger dependence on node degrees compared to its bias-aware variant, providing evidence that ROBUST (bias-aware) successfully reduces the influence of research bias and degree-based shortcuts.

Interestingly, except for DOMINO, the AMIMs exhibit increased node-degree dependence with larger networks, which is statistically significant for ROBUST, RWR, and 1st Neighbors (Fig. 6B). This suggests that while the overall impact of node-degree bias on the AMIMs is only moderate, it becomes increasingly relevant for larger networks.

## 3 Discussion

Our pipeline provides access to a diverse collection of disease module discovery algorithms within the most comprehensive evaluation framework to date. Applied to 50 input combinations, the pipeline revealed characteristic strengths of the included methods while also highlighting limitations, such as strong dependence on the input network and only moderate performance in seed rediscovery analyses.

In our demonstration, each AMIM showed distinct strengths and limitations that should be considered for a specific use case. Our analyses further underscored the critical influence of the input network choice. Although our findings do not support a specific network recommendation, we observed that larger networks are more frequently associated with undesirable behaviors when combined with certain AMIMs. For those, we anticipate smaller networks to yield more reliable results. To guide users in selecting the most suitable approach, we summarize the outcomes of our analyses in Table 1.

**Table 1:** Summary of the AMIM properties revealed through the analysis performed to demonstrate the pipeline.

	DOMINO	ROBUST	ROBUST (bias-aware)	DIAMOnD	RWR	1st Neighbors
Approach	Clustering	Connecting the seeds	Connecting the seeds	Expanding from the seeds	Expanding the seeds until connected	Expanding from the seeds
Designed for multiple, disconnected mechanisms?	✔	✗	✗	✔	✗	✔
Retains all seed nodes in the module? (Fig. 2C)	✗	Yes, if part of the input network’s LCC	Yes, if part of the input network’s LCC	✔	Yes, if part of the input network’s LCC	✔
Adds nodes distant from the seed nodes? (Fig. 2D)	Rarely adds 2nd neighbors	Sometimes adds 2nd and 3rd neighbors	Frequently adds distant nodes	Frequently adds 2nd and 3rd neighbors	Sometimes adds 2nd and 3rd neighbors	No, only direct interactors
Modules are specific to the seed set? (Fig. 3A)	✔	✔	✔	Only for some seed sets	Only for some seed sets	Only for some seed sets
Modules are specific to the seed set (only considering added nodes)? (Fig. 3B)	Only for some seed sets	✗	✗	✗	✗	✗
Robust to leave-one-out perturbations? (Fig. 4)	✗	✔	✔	✔	✔	✔
Good at rediscovering left-out-seeds? (Fig. 5A)	✗	✗	✗	✗	Moderate	✔
Good at rediscovering left-out-seeds (considering module size)? (Fig. 5B)	Moderate	Moderate	✗	✔	✔	✗
Effect of larger networks	Worse robustness; worse normalized seed rediscovery	Smaller modules; worse normalized seed rediscovery; stronger degree dependence	Worse robustness; worse normalized seed rediscovery	—	Smaller modules; stronger degree dependence	Larger modules; worse normalized seed rediscovery; stronger degree dependence

We found that the AMIMs in our pipeline produced comparable modules when a seed gene is left out, but struggled to consistently recover the left-out seed and to produce disease-specific modules independent of the input network choice. This suggests that the AMIMs cannot always reliably distinguish between truly disease-relevant nodes and background noise in the underlying networks. In the future, we plan to leverage our modular pipeline design to integrate additional AMIMs (Wang and Loscalzo, 2018; Buzzao *et al*. 2022) and evaluate whether alternative algorithms can improve performance. However, the lack of performance in these categories could also stem from excessive noise or incompleteness in the input networks or seed sets (Stumpf and Wiuf, 2010; Guney and Oliva, 2014), which may not be resolved by algorithmic improvements alone. For both input types, there is an inherent trade-off between increased false-positive rates in larger network and seed set sizes and increased false-negative rates in smaller ones. All AMIMs currently implemented in the pipeline use binary seed weights and network edges, which we focused on due to their flexibility, simplicity, and performance in previous studies (Levi *et al*. 2021). A possible way to address this trade-off between input size and accuracy in the future would be to integrate methods that use continuous node weights (Reyna *et al*. 2018), or weighted network edges, for example, by using edge-dependent transition probabilities in an RWR approach, enabling direct representation of confidence levels in disease associations or protein interactions. Furthermore, the input networks provided through our pipeline interface are context-agnostic and do not account for tissue-specific variations in PPIs. While users can already provide custom, context-specific networks, we plan to add functionality that automatically filters PPIs based on genes or proteins expressed in a specified tissue of interest, leveraging expression databases.

While the evaluation procedures in our pipeline provide insights into the reliability of the inferred disease modules, these should be considered only as indicative evidence, since a complete ground truth would be required to assess AMIM performance definitively. Moreover, certain evaluation steps may be subject to research bias and circular reasoning. For example, seed sets often consist of well-studied disease genes or proteins with many known PPIs, making them easier to rediscover using network-based approaches. Additionally, these genes or proteins may have already been analyzed together in the context of specific diseases or pathways, potentially biasing the assessment of functional coherence or pathway enrichment analyses. The underperformance of the research-bias-aware implementation of ROBUST in these analyses further supports the notion that evaluation procedures relying on prior knowledge are susceptible to such biases. Furthermore, not every evaluation step returned conclusive results, e.g., the functional coherence analysis using DIGEST (Adamowicz *et al*. 2022) did not reveal meaningful patterns across AMIMs (see Supplementary Note 2, available as supplementary data at *Bioinformatics* online). The integrated over-representation analysis indicates that, in some cases, disease modules enhance the enrichment of disease-associated pathways compared to seed genes alone (see Supplementary Note 3, available as supplementary data at *Bioinformatics* online). However, the current analysis is limited in that it considers disease modules only as a whole. In a future pipeline release, we plan to perform the analysis specifically on the added module nodes to gain more detailed insights into their individual contributions.

Limitations of our pipeline itself include the current inability to explore different tool parameter configurations or tunings within a single run. Additionally, the seed-set perturbation strategy supports only a basic leave-one-out approach, which we plan to extend by options to omit multiple seed nodes simultaneously or add random distractor genes to assess robustness. Furthermore, algorithmically inferred disease modules often require curation by biomedical experts, which cannot be fully automated. To facilitate this process, we provide Drugst.One (Maier *et al*. 2024) export links, which, together with its recent extension, Drugst.One DREAM (Spindler *et al*. 2026), supports manual module refinement.

## 4 Methods

### 4.1 Module topology and overlap

Let N=(V,E) be the input network with node set *V* and edge set *E*, *S* the seed node set with S⊆V, and M(S,N)⊆V the set of nodes in a disease module inferred with seeds *S* and network *N*. All topological measures for a disease module are calculated on its induced subnetwork N[M(S,N)] with node set M(S,N) and edge set consisting of all edges in *E* that connect two nodes included in M(S,N).

The maximum distance from any added node *a* to its nearest seed *s* is calculated as $\max_{a\in M(S,N)\setminus S,s\in S}d\mathrm{ist}(a,s)$, where dist(a,s) is the length of the shortest path between nodes *a* and *s* in the subnetwork induced by the node set M(S,N). A pseudo diameter (heuristic estimate of the longest shortest path between any two nodes) is calculated using the *graph-tool* library (https://graph-tool.skewed.de/, v2.77).

The pipeline calculates pairwise Jaccard similarities between two module node sets *A* and *B*, i.e., $J_{A,B}=\frac{\left|A\cap B\right|}{\left|A\cup B\right|}$. To focus only on added nodes, the same measure is computed on A∖S and B∖S.

### 4.2 Input perturbation

Given an initial seed set $S=\{s_{1},s_{2},\ldots ,s_{n}\}$, the pipeline generates perturbed sets $S_{-i}=S\setminus \{s_{i}\}$ by systematically removing each seed node $s_{i}$. For each perturbed set $S_{-i}$ and corresponding network *N*, a module with node set $M(S_{-i},N)$ is computed and compared to the original node set of the original module M(S,N).

The robustness for each perturbation run is measured through the Jaccard similarity, i.e., $J_{i}=\frac{\left|M(S,N)\cap M(S_{-i},N)\right|}{\left|M(S,N)\cup M(S_{-i},N)\right|}$, and summarized across runs using the mean.

The rediscovery rate (Maier *et al*. 2023) is calculated as the fraction of omitted seeds that are reincluded in the corresponding run, i.e., $R=\frac{1}{n}\sum_{i=1}^{n}d_{i}$, where $d_{i}$ is 1 if $s_{i}\in M(S_{-i},N)$ and 0 otherwise. The rediscovery rate is normalized to account for the size of the original module, i.e., $R_{\mathrm{norm}}=\frac{R}{\left|M(S,N)\right|}$.

A network *N* defined through a set of nodes *V* and edges *E* is rewired by iteratively selecting four nodes a,b,c,d∈V with (a,b)∈E and (c,d)∈E, and replacing the edges with (a,d) and (b,c), provided that this operation does not introduce self-loops or duplicate edges (Viger and Latapy, 2016). The network rewiring is performed using the *graph-tool* function *random_rewire* with “constrained-configuration” as model and 100 full sweeps over all edges. The number of perturbed networks *m* defaults to 100. Modules with node sets $M(S,N_{j})$ are inferred for every perturbed network $N_{j}$. The perturbation impact is again measured using Jaccard similarity, i.e., $J_{j}=\frac{\left|M(S,N)\cap M(S,N_{j})\right|}{\left|M(S,N)\cup M(S,N_{j})\right|}$, and summarized across all perturbed networks using the mean.

### 4.3 Pipeline demonstration

To demonstrate the pipeline, disease-associated genes from five complex diseases were used as seed sets. These genes were obtained from DisGeNET (https://www.disgenet.com/, v24.3) (Piñero *et al*. 2020) (see Table S2). The demonstration utilized all ten networks integrated into the pipeline (see Table S1) and was executed in a single run (see Supplementary Note 4, available as supplementary data at *Bioinformatics* online).

The pipeline was executed using Nextflow (v24.10.05) on a high-performance computing cluster running Ubuntu (v22.04.5), using approximately 2,300 CPU hours. Software dependencies were managed using Singularity (v3.8.7). Downstream analyses and visualizations were performed in Python (v3.12) using the *seaborn* package (https://github.com/mwaskom/seaborn, v0.13). Correlations between continuous variables and the corresponding p-values were computed using Pearson’s correlation coefficient, as implemented in *scipy.stats.pearsonr* (https://github.com/scipy/scipy, v1.14). Linear regression lines were fitted and plotted using *seaborn’s lmplot* function. Hierarchically clustered heatmaps were generated with *seaborn’s clustermap* function using the “ward” linkage method. The order of columns and rows was randomly shuffled prior to clustering to eliminate potential biases arising from the initial ordering.

## 5 Data and code availability

Pipeline code: GitHub (https://github.com/nf-core/diseasemodulediscovery) and Zenodo (https://doi.org/10.5281/zenodo.17674968); Analysis scripts for the pipeline demonstration: GitHub (https://github.com/REPO4EU/modulediscovery_demonstration) and Zenodo (https://doi.org/10.5281/zenodo.19186412); Input network preparation code: GitHub (https://github.com/REPO4EU/network_preparation) and Zenodo (https://doi.org/10.5281/zenodo.17674988); Prepared input networks: Zenodo (https://doi.org/10.5281/zenodo.15049754); Results of the pipeline demonstration run: Zenodo (https://doi.org/10.5281/zenodo.17536307).

## Supplementary Material

btag223_Supplementary_Data

## Supplementary information

Supplementary figures and tables are available at *Bioinformatics* online.
