# Supplementary material for: Inferring and evaluating network medicine-based disease modules with nextflow
Source: Bioinformatics. 2026 Jul 7;42(Suppl 1):btag223. doi: 10.1093/bioinformatics/btag223 (PMC13340264; doi:10.1093/bioinformatics/btag223)
Supplement: btag223_Supplementary_Data [file btag223_supplementary_data.pdf]

Supplement: Inferring and Evaluating Network Medicine-Based Disease Modules with Nextflow

Johannes Kersting[1], Chloé Bucheron[2,3,4], Lisa M. Spindler[1], Joaquim Aguirre-Plans[5], Quirin Manz[1], Tanja Pock[1], Mo Tan[1], Fernando M. Delgado-Chaves[6], Cristian Nogales[2,4], Harald H. H. W. Schmidt[7], Jörg Menche[2,4,8,9], Andreas Maier[6], Jan Baumbach[6], Emre Guney[5], Markus List[1,10,*]

[1]Data Science in Systems Biology, TUM School of Life Sciences, Technical University of Munich, Freising, Germany
[2]Max Perutz Labs, Department of Structural and Computational Biology, University of Vienna, Vienna, Austria
[3]Vienna Biocenter PhD Program, a Doctoral School of the University of Vienna and the Medical University of Vienna, Vienna, Austria
[4]Ludwig Boltzmann Institute for Network Medicine at the University of Vienna, Vienna, Austria
[5]Discovery and Data Science (DDS) Unit, STALICLA SL, Barcelona, Spain
[6]Institute for Computational Systems Biomedicine, University of Hamburg, Hamburg, Germany
[7]Department of Pharmacology and Personalised Medicine, Maastricht University, Maastricht, The Netherlands
[8]Faculty of Mathematics, University of Vienna, Vienna, Austria
[9]CeMM Research Center for Molecular Medicine of the Austrian Academy of Sciences, Vienna, Austria
[10]Munich Data Science Institute (MDSI), Technical University of Munich, Garching, Germany

*Corresponding author: markus.list@tum.de

Keywords:
network medicine, disease modules, drug repurposing, pipeline, Nextflow

Supplementary Notes

Supplementary Note 1: Effect of method-specific parameters on disease module topology

The topological properties of disease modules inferred by different AMIMs were obtained using default parameter settings (see **Supplementary Methods**). Adjusting these parameters can directly affect several of the investigated properties. For DIAMOnD, module size is determined by the number of added nodes (n), and the seed weight (α) will influence explorativeness. In DOMINO, module size depends on the confidence thresholds (`slices_threshold` and `module_threshold`). For ROBUST, size is affected by the node inclusion prize (α) and the tree fraction threshold (θ). In its bias-aware variant, the study-bias penalty (γ) can be reduced to yield modules closer to the original implementation. For RWR, a trade-off between local and global exploration of the seed's neighborhood is determined by the restart probability r.

Supplementary Note 2: Function-based module coherence depends on the individual case

The tool DIGEST was specifically developed to evaluate disease modules. It is based on the assumption that the genes within a module should be functionally coherent, i.e., involved in similar biological processes, represented through gene set annotations from Gene Ontology (GO) [1,2], including Biological Process (GO.BP), Cellular Component (GO.CC), and Molecular Function (GO.MF), as well as the Kyoto Encyclopedia of Genes and Genomes (KEGG) [3]. The pipeline integrates DIGEST in two different modes. The reference-free mode assesses the internal functional coherence between all module nodes, while the reference-based mode compares the functional coherence between the seed nodes and the added nodes.

In the reference-free mode (**Figure S9A**), the raw seed genes already exhibit strong functional coherence for GO.BP and KEGG, with coherence decreasing upon the inclusion of additional nodes. For GO.CC, only DIAMOnD and the 1st Neighbors approach improve upon the seed gene baseline. For GO.MF, significant results are observed only sporadically. In the reference-based mode (**Figure S9B**), the 1st Neighbors approach achieves the highest coherence across gene set sources. By contrast, ROBUST (bias-aware) yields modules with the lowest coherence in both modes, likely due to its inclusion of more distant genes (**Figure 2D**), which may be less functionally related. Overall, module coherence appears to depend strongly on the specific case, mode, and gene set source, without consistent patterns across different seed sets or input network configurations (**Figure S10** and **Figure S11**).

Supplementary Note 3: Disease modules can enhance enrichment of disease-associated pathways compared to the seed genes alone

A common strategy for assessing the biological relevance of inferred disease modules is to test for enrichment of predefined gene sets, such as biological pathways, known disease genes, or Gene Ontology (GO) terms, using over-representation analysis [4–6]. The results provide insights into the biological processes captured by the module and its reliability by comparing the findings to user expectations. To support this analysis, the pipeline reports enriched gene sets by running g:Profiler [7] (see **Supplementary Methods**).

To this end, we performed a focused analysis of selected KEGG [3] disease pathways corresponding to the diseases under study. While Huntington's disease (HD) and amyotrophic lateral sclerosis (ALS) can be directly matched to specific KEGG pathways, we mapped ulcerative colitis (UC), Crohn's disease (CD), and lung adenocarcinoma (LUAD) to their closest available parent terms.

Figure S12 summarizes the KEGG pathways most frequently enriched for each disease. The matched KEGG disease pathways consistently rank among the top entries. Although this is expected, given that enrichment analysis was performed on all module nodes, including the disease-associated seed genes, it nonetheless confirms the disease relevance of the seed genes using an additional resource (KEGG) and demonstrates that this association is preserved in the inferred modules.

Further, examining the enrichment significance of the best-matching KEGG disease pathway (**Figure S13**) shows that some modules show stronger associations than the seed genes alone. In particular, for HD, the inclusion of first neighbors consistently improves the association with the corresponding KEGG pathway. Similarly, modules derived from one of the STRING [8] networks often exhibit more significant enrichment than the seed-only sets. As STRING integrates multiple evidence channels to score protein-protein interactions, including pathway annotations, this may explain the closer alignment with KEGG-based enrichment results.
While this analysis provides insight into the biological processes captured by the disease modules, comparing enrichment significance between full modules and seed-only sets allows only limited conclusions about the specific contribution of the added genes. In future versions of the pipeline, we plan to extend this functionality by performing over-representation analysis on the added genes alone (i.e., excluding seed genes) to support a more fine-grained assessment of their contribution.

Supplementary Note 4: The command that was used to run the pipeline demonstration

```
nextflow run nf-core/diseasemodulediscovery \
-r 10d49e1a3808f1046c181d1e6b7dac1481f2bdb5 \
-profile daisybio,singularity,keep_work \
--id_space symbol \
--seeds
../../data/seeds/ALS.tsv,../../data/seeds/CD.tsv,../../data/seeds/HD.tsv
,../../data/seeds/LUAD.tsv,../../data/seeds/UC.tsv \
--network
string_min900,string_min700,string_physical_min900,string_physical_min70
0,biogrid,hippie_high_confidence,hippie_medium_confidence,iid,nedrex,ned
rex_high_confidence \
--run_seed_permutation \
--run_network_permutation \
--outdir results
```

Supplementary Methods

Implementation

The pipeline is implemented using Nextflow [9] with its DSL2 extension, which supports a modular design for improved maintainability, configurability, and future expandability. Nextflow manages the execution of all pipeline steps and parallelizes processes where possible. It caches the results of individual steps, enabling resumption from the last successful point in case of failure. This also allows for running specific parts of the pipeline and incorporating additional steps later without needing to recompute previous results. Software dependencies are automatically deployed through Docker or Singularity [10], requiring the user to install only Nextflow and a compatible container runtime. Nextflow pipelines are highly portable, allowing them to run on HPC clusters as well as various cloud computing platforms.

Our pipeline is part of the nf-core project [11] and uses the nf-core template, adhering to best practices for code structure and documentation. Continuous integration (CI) tests monitor the pipeline development, and an included test dataset enables users to easily verify a successful installation.

Custom analysis scripts are written in Python (v3.12) and are centered around the *graph-tool* library [12] (https://graph-tool.skewed.de/, v2.77) for network analysis and its binary GT file format and for efficiently passing graph data between processes. These dependencies are deployed via a shared container (https://github.com/REPO4EU/modulediscovery_python_dependencies). Most external tools are run in dedicated containers for better modularity and to prevent software version conflicts.

Input

The main inputs to the pipeline are a text file containing the seed nodes (one per line) and a network file, which can be in CSV, GT, GraphML, or DOT format. It is also possible to provide multiple files for each input type. In this case, the pipeline can either run on all possible input combinations or on specific pairs defined in a sample sheet. Seed files are filtered to include only nodes present in the corresponding network file. If multiple networks are provided, the pipeline generates a separately filtered seed file for each network.

Some tools further require specifying the ID space of the input. Supported ID spaces are HGNC Symbols [13], Entrez IDs [14], or Ensembl IDs [15] for genes and UniProt accession numbers (AC) [16] for proteins.

Available networks

Instead of supplying their own input network, users can select from a variety of widely used human PPI networks, including STRING [17], BioGRID [18], HIPPIE [19], IID [20], and NeDRex [21]. For several of the sources, we provide multiple subsets, resulting in a total of ten network options from five databases. For STRING, users can choose between networks that include or exclude non-physical functional interactions, each available with two

confidence thresholds (score > 0.9 or > 0.7). For HIPPIE, both medium- and high-confidence versions are available, based on the score cutoffs of 0.63 and 0.73, respectively, as defined on the HIPPIE website (https://cbdm-01.zdv.uni-mainz.de/~mschaefer/hippie/information.php). For NeDRex, we only included experimentally validated interactions between reviewed proteins. We provide a standard and a high-confidence version, the latter filtered by a method score greater than 13.5 [22].

The networks were downloaded from their respective primary sources, using UniProt accession numbers (ACs) as ID space whenever available. HIPPIE and STRING did not provide UniProt ACs directly; instead, they used UniProt entry names and Ensembl protein IDs, respectively. These identifiers were converted to UniProt ACs using UniProt's official ID mapping file (https://ftp.uniprot.org/pub/databases/uniprot/current_release/knowledgebase/idmapping/by_organism/HUMAN_9606_idmapping_selected.tab.gz, accessed on 18.03.2025).

The mapping file provides a one-to-one correspondence between UniProt entry names and UniProt ACs, but may result in a many-to-many mapping between Ensembl protein IDs. When an original identifier mapped to multiple UniProt ACs, we created a separate network node for each resulting UniProt AC and assumed each to interact with all partners of the original identifier. Conversely, when multiple original identifiers mapped to the same UniProt AC, the corresponding nodes were merged.

The resulting UniProt AC-based networks were subsequently mapped to the other supported ID spaces. Ensembl and Entrez gene IDs were obtained using the same UniProt mapping file, while HGNC symbols were retrieved using the *mygene* Python package (https://github.com/biothings/mygene.py, v3.2.2), as they were not included in the UniProt mapping file. Multi-mapper IDs were handled as outlined above. Duplicate edges and self-loops were removed from all networks.

The code for downloading, parsing, and mapping all networks is available at https://github.com/REPO4EU/network_preparation. The process is fully automated, and both download sources and parameters can be configured via a settings file to easily add or modify data sources for future releases.

To use the available networks, instead of a file path, the user can specify a keyword to automatically load the network (see **Table S1**).

Integrated AMIMs

The pipeline currently incorporates six disease module discovery methods, chosen based on their popularity, code availability, and ease of integration. Default parameter values are derived from each method's recommended or standard settings and can either be configured directly through pipeline parameters or modified by supplying custom command-line options to the respective tools. The included methods are:

DOMINO (Discovery of Modules In Networks using Omic) [5] begins by partitioning the network into disjoint slices using Louvain clustering [23], selecting those enriched for seed

nodes based on a hypergeometric test. The selected slices are refined by solving the Prize Collecting Steiner Tree (PCST) problem [24] and are then further subdivided into putative modules, each containing no more than ten nodes. These modules are again tested for seed enrichment using a hypergeometric test. DOMINO outputs a flexible number of modules, which may belong to the same or to different connected components. In the pipeline, all returned modules are merged into a single module by taking their union. Each node is annotated with an additional identifier indicating its original DOMINO module. DOMINO (https://github.com/Shamir-Lab/DOMINO, v1.0.0) is deployed in the pipeline via its available biocontainer (https://quay.io/repository/biocontainers/domino). The method provides parameters to adjust the significance thresholds for slices (`slices_threshold`) and putative modules (`module_threshold`), which default to 0.3 and 0.05, respectively.

The **DIAMOnD** (DIseAse MOdule Detection) algorithm [4] iteratively expands an initial set of seed nodes by adding one node at a time. At each step, it selects the node with the highest connectivity significance to the current seed set, computed using a hypergeometric test. This process continues until a predefined number of nodes have been added. DIAMOnD only returns a list of added nodes, so the pipeline combines these with the seed nodes to produce the final output module. The result is a single disease module, which may consist of one or multiple connected components. DIAMOnD (https://github.com/dinaghiassian/DIAMOnD) is deployed in the pipeline via a custom container (https://hub.docker.com/r/djskelton/diamond) originally created for the NeDRex platform. The total number of added nodes n and the weight assigned to the initial seeds α are configurable via parameters and default to 200 and 1.0, respectively, in accordance with the authors' recommendations.

ROBUST (robust disease module mining via enumeration of diverse prize-collecting Steiner trees) [6] repeatedly connects seed genes by solving the PCST problem. In each iteration, nodes included in previous solutions are penalized, reducing their likelihood of being selected again. The final disease module comprises nodes that have appeared in a sufficient number of these solutions to increase robustness. It may consist of one or multiple connected components. ROBUST (https://github.com/bionetslab/robust) is deployed in the pipeline via a custom container (https://hub.docker.com/r/djskelton/robust) originally created for the NeDRex platform. The initial value of integrating non-seed nodes α, value reduction factor β, number of PCST n, and the fraction of PCST runs a node must appear in to be included in the final module τ can all be configured via parameters. By default, these are set to $(\alpha, \beta, n, \tau) = (0.25, 0.9, 30, 0.1)$, based on the authors' recommendations.

ROBUST (bias-aware) [25] adopts the same strategy as ROBUST but increases the edge costs for nodes frequently used as baits in PPI detection experiments. This penalization is designed to counteract study bias in current PPI networks [26]. ROBUST (bias-aware) (https://github.com/bionetslab/robust_bias_aware, v.0.0.1) is deployed in the pipeline via its available biocontainer (https://quay.io/repository/biocontainers/robust-bias-aware). The method provides the same parameters as ROBUST, along with an additional γ parameter (default: 1.0), which can be used to adjust the study-bias edge penalty.

RWR (Random Walk with Restart) [27] models signal diffusion on a network by simulating a walker that moves randomly between connected nodes but, with a restart probability r,

returns to the seed nodes. This parameter balances global exploration of the interactome with local focus around the seeds, and the resulting steady-state probabilities provide a ranking of proteins by their relevance to the seeds. The size of the disease module is determined by including nodes ranked by the RWR until all seeds are connected, which will result in a module consisting of exactly one connected component. RWR is deterministically implemented (no simulation). We set r to 0.8 to have an effective trade-off between local and global exploration of the seeds' neighborhood [28]. The method can scale the nodes' visiting probability by the square root of their degree using the `scaling` parameter (default: False) and allows the use of the symmetrical Markov matrix with the `symmetrical` parameter (default: False).

1st Neighbors [29] includes every network node that directly interacts with at least one seed, resulting in a module that may contain one or more connected components. The pipeline implements this approach using the *graph-tool* library.

Additionally, the pipeline integrates a pseudo-AMIM that outputs only the seed nodes along with the edges connecting them within the input network. Filtering the seed genes that are not present in the input network, this configuration serves as a baseline for comparison with other AMIMs and is referred to as "**Only seeds**" throughout this manuscript.

Biological module evaluation

Over-representation analysis is performed using g:Profiler through its R package *gprofiler2* (https://cran.r-project.org/web/packages/gprofiler2/index.html, v0.2.2) [7], which is integrated into the pipeline via the corresponding nf-core module (https://nf-co.re/modules/gprofiler2_gost/). The pipeline performs over-representation analysis using module nodes as the foreground and all network nodes as the background. By default, it considers gene sets from Gene Ontology (GO) [1,2], WikiPathways [30], Reactome [31], and KEGG [3]. P-values are corrected for multiple testing using g:Profiler's tailored method, g:SCS [32].

Functional coherence of the modules is assessed using DIGEST [33] (https://github.com/bionetslab/digest, v0.2.16), which the pipeline integrates via its biocontainer (https://quay.io/repository/biocontainers/biodigest). The pipeline runs DIGEST in two modes: a reference-free mode (`mode="subnetwork"`), which evaluates the functional coherence of all nodes within a module, and a reference-based (`mode="subnetwork-set"`) mode, which assesses the coherence between the seed nodes and the nodes added during module construction. Both modes use Jaccard similarity as a distance metric and rely on the pipeline input network(s) to generate 1,000 random modules for perturbation-based significance testing.

Annotation and BioPAX format

To characterize the inferred disease modules functionally, we perform an automated annotation step using information from NeDRexDB [21], a knowledge graph integrating a range of primary databases for network medicine-based drug repurposing. The annotated

modules are represented through the standardized BioPAX [34] Level 3 format. The publicly available NeDRex API is queried based on the genes or proteins included in the disease module. If the module nodes are represented by gene-level identifiers, the corresponding protein products are annotated. Conversely, if protein-level identifiers are used, the associated coding genes are annotated. Disorders are linked to genes according to known associations. For proteins targeted by drugs, the corresponding drugs are retrieved together with their known side effects. Based on the obtained drugs and disorders, indications and contraindications of drugs for disorders are incorporated. Moreover, cellular components associated with the proteins are integrated based on GO annotations [1,2]. The *pybiopax* Python package (https://github.com/gyorilab/pybiopax, v0.1.4) [35] is used to parse and process BioPAX data. Since BioPAX Level 3 does not natively support entities for disorders and side effects, these are included as external references.

Drugst.One export and drug prioritization

For each inferred disease module, the pipeline generates a hyperlink enabling interactive exploration via the network medicine web tool Drugst.One [36]. These links are included in the pipeline report. Additionally, the associated Python package, *drugstone* (https://github.com/drugst-one/python-package, v0.4.5), is used to identify potential drug candidates that target module nodes. To prioritize compounds, the pipeline integrates three network-based ranking algorithms (see Figure 1B): degree centrality, which ranks compounds by the number of their targets within the module; harmonic centrality, which considers the average shortest distance from each compound to all module nodes; and TrustRank, ranking compounds based on network propagation [37]. Further details on these methods can be found in the supplementary material of the Drugst.One publication [36].

Network visualizations and pipeline report

Visual network representations of the inferred modules (both with and without assigned drugs) are generated using the graph-tool package and provided in PNG, SVG, and PDF formats. Additionally, interactive HTML visualizations are created with the pyvis package (https://github.com/WestHealth/pyvis, v0.3).

The pipeline results are summarized through an HTML report created with MultiQC (https://github.com/MultiQC/MultiQC, v1.27) [38]. MultiQC is a standard component of many nf-core pipelines, providing overviews of tool outputs, software versions, and execution commands. As MultiQC does not natively support the tools in our pipeline, they are integrated using MultiQC's functionality to incorporate custom content.

Supplementary Tables

Table S1: *Overview of PPI networks accessible via the pipeline interface. For sources without versioning, the query date is provided. Node and edge counts are based on UniProt AC IDs.*

Keyword	Version	Nodes	Edges	Description
`string_min900`	v12.0	11,971	93,559	Human PPI network obtained from STRING, including physical and functional interactions with a score greater than 0.9.
`string_min700`	v12.0	15,788	224,045	Human PPI network obtained from STRING, including physical and functional interactions with a score greater than 0.7.
`string_physical_min900`	v12.0	7,722	34,141	Human PPI network obtained from STRING, including physical interactions with a score greater than 0.9.
`string_physical_min700`	v12.0	10,465	78,878	Human PPI network obtained from STRING, including physical interactions with a score greater than 0.7.
`biogrid`	4.4.242	18,101	865,553	Human PPI network obtained from BioGRID.
`hippie_high_confidence`	v2.3	13,246	112,202	Human PPI network obtained from HIPPIE, including only high-confidence interactions with a score greater than 0.73.
`hippie_medium_confidence`	v2.3	16,613	637,499	Human PPI network obtained from HIPPIE, including only interactions with a score greater than 0.63.
`iid`	2025-03-18	19,598	1,202,716	Human PPI network obtained from IID.
`nedrex`	2025-03-18	18,718	935,139	Human PPI network queried from NeDRexDB, including only experimentally validated interactions.
`nedrex_high_confidence`	2025-03-18	12,827	95,944	Human PPI network queried from NeDRexDB, including only experimentally validated interactions with a score greater than 13.5.

Table S2: *Overview of the seed gene sets used for the pipeline demonstration.*

**Abbreviation**	**Full name**	**DisGeNET term**	**Number of genes**
HD	Huntington’s disease	Huntington Disease, C0020179	40
UC	Ulcerative colitis	Ulcerative Colitis, C0009324	76
CD	Crohn’s disease	Crohn's disease of large bowel, C0156147	78
ALS	Amyotrophic lateral sclerosis	Amyotrophic Lateral Sclerosis, C0002736	127
LUAD	Lung adenocarcinoma	Adenocarcinoma of lung, C0152013	280

Supplementary Figures

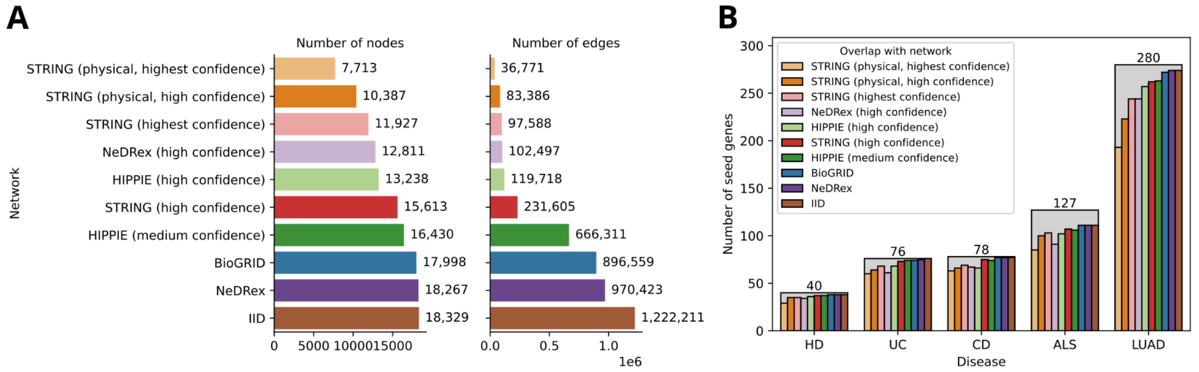

Figure S1: *Inputs for the pipeline demonstration. (**A**) Number of nodes and edges across the ten input PPI networks. (**B**) Size of the five disease gene sets, shown as the number of seed nodes (gray bars with annotations), and their overlaps with the node sets of the input networks (colored bars).*

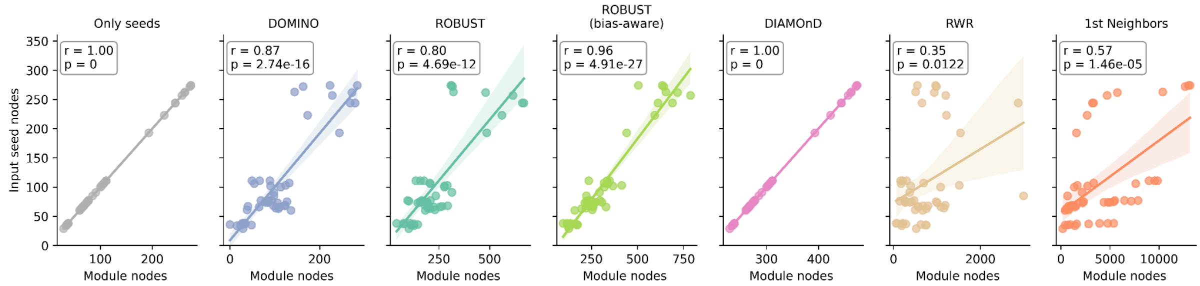

Figure S2: *Correlation between the number of module nodes and the number of seed nodes used. r indicates the Pearson correlation and p the corresponding p-value.*

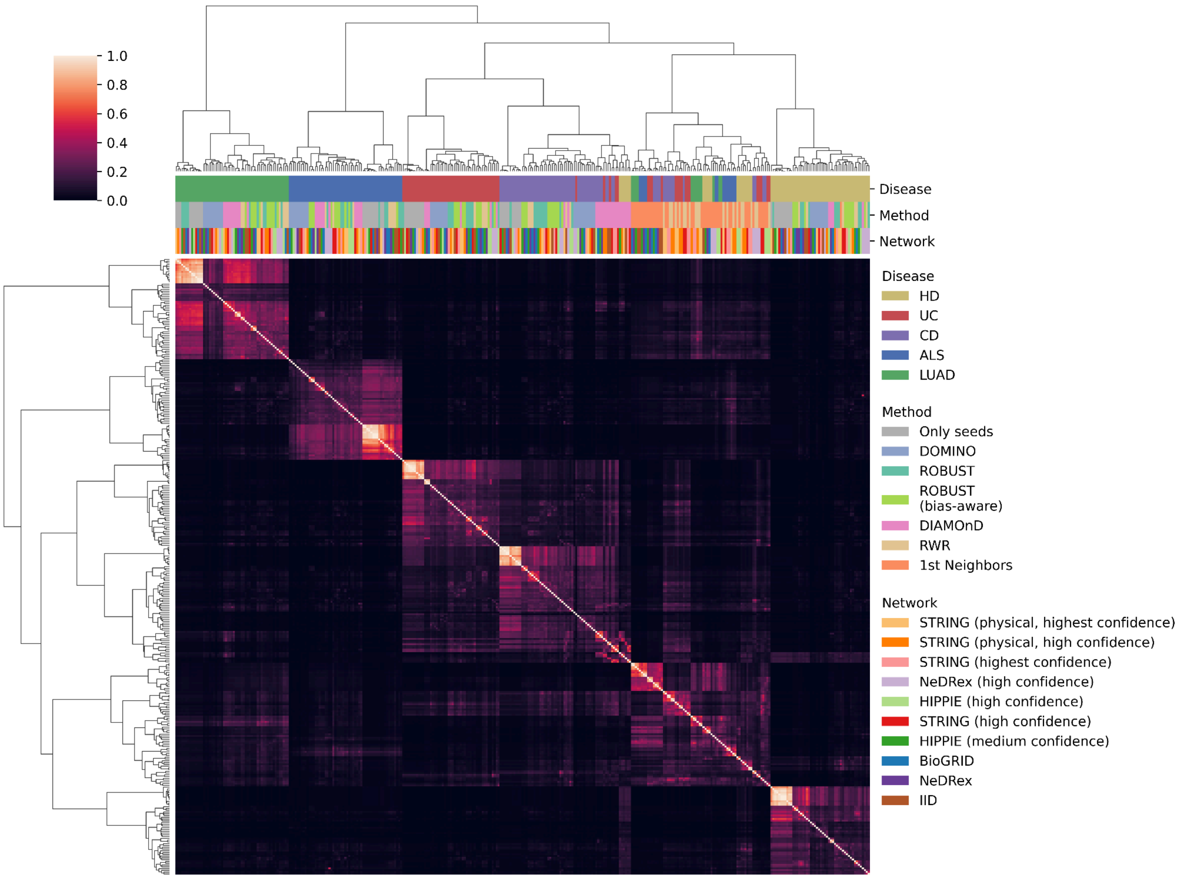

Figure S3: *Heatmap and hierarchical clusterings based on the pair-wise node set similarities (measured through the Jaccard similarity) of modules inferred using different diseases, networks, and methods. All module nodes are considered.*

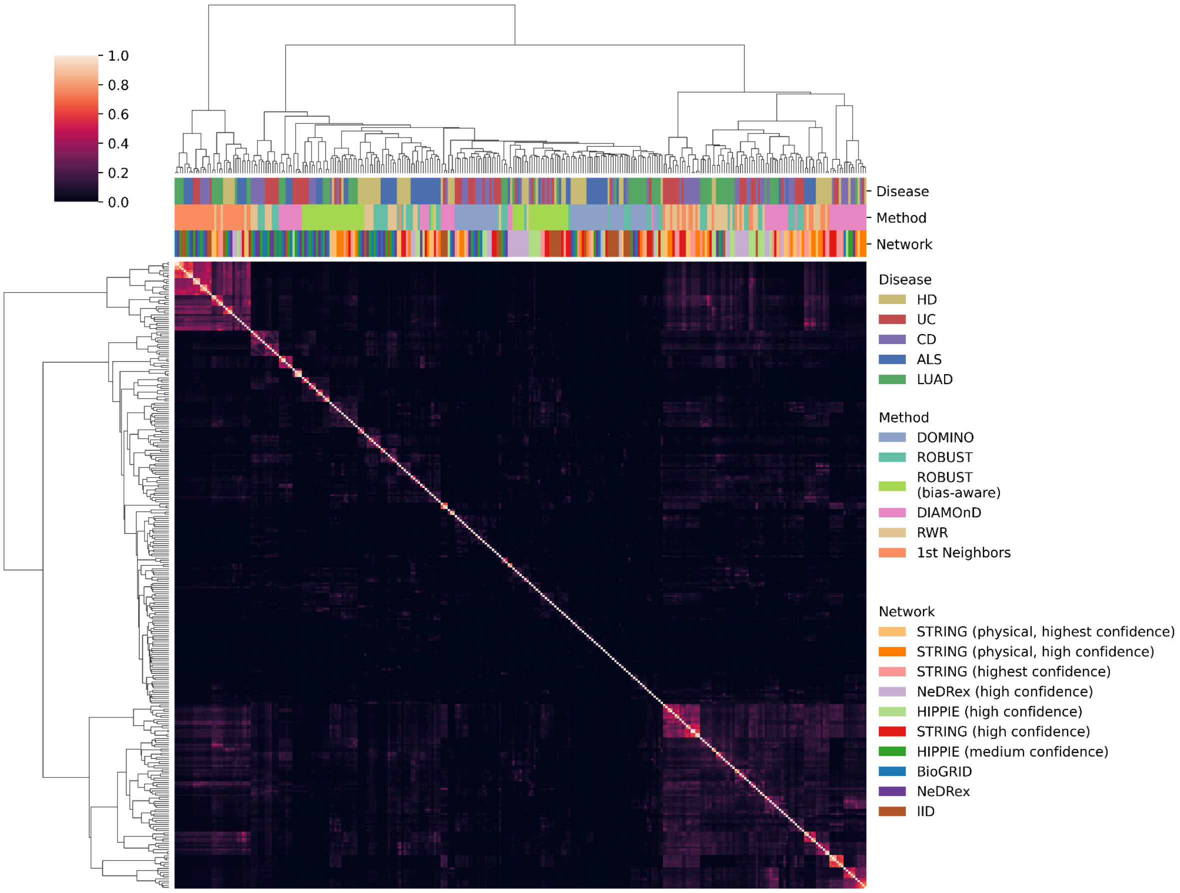

Figure S4: *Heatmap and hierarchical clusterings based on the pair-wise node set similarities (measured through the Jaccard index) of modules inferred using different diseases, networks, and methods. Only added nodes (no seed nodes) are considered for the overlap calculation.*

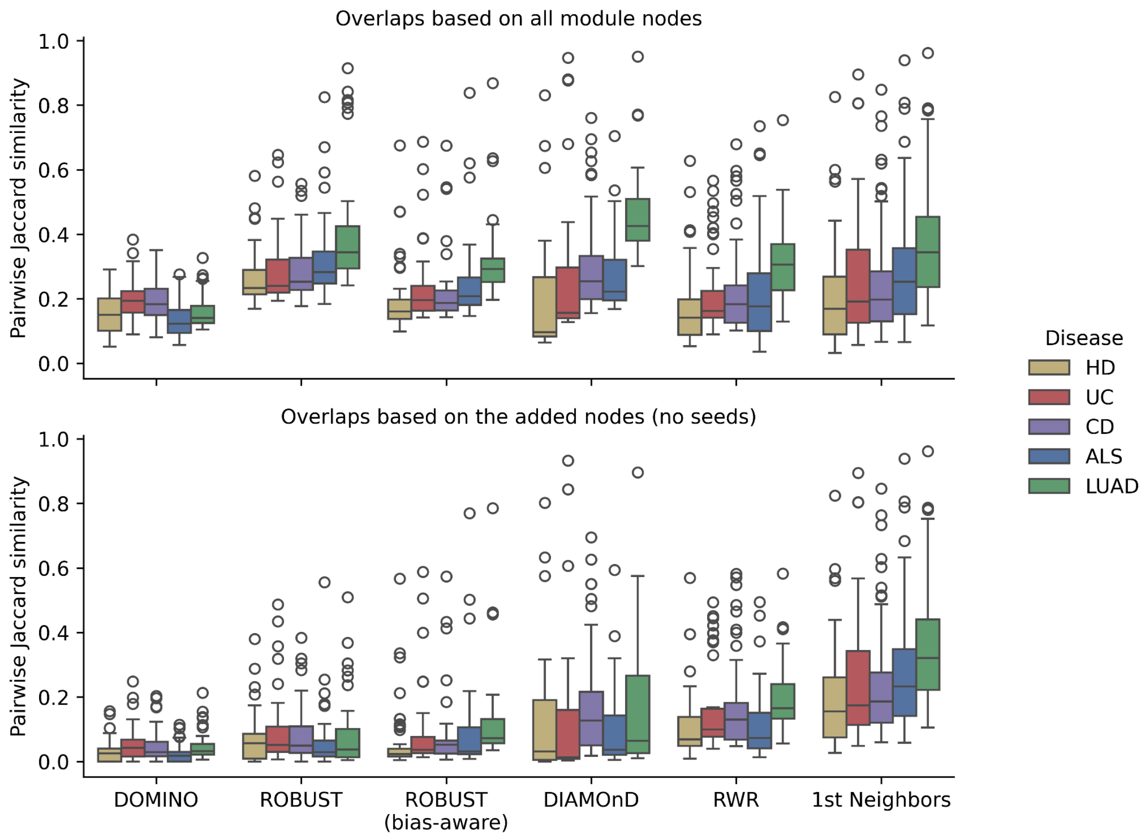

Figure S5: *Distributions of the pair-wise Jaccard similarities between the node sets of disease modules inferred using the same seed set and AMIM, but different input networks. Top: considering all module nodes. Bottom: only considering added nodes (no seed nodes).*

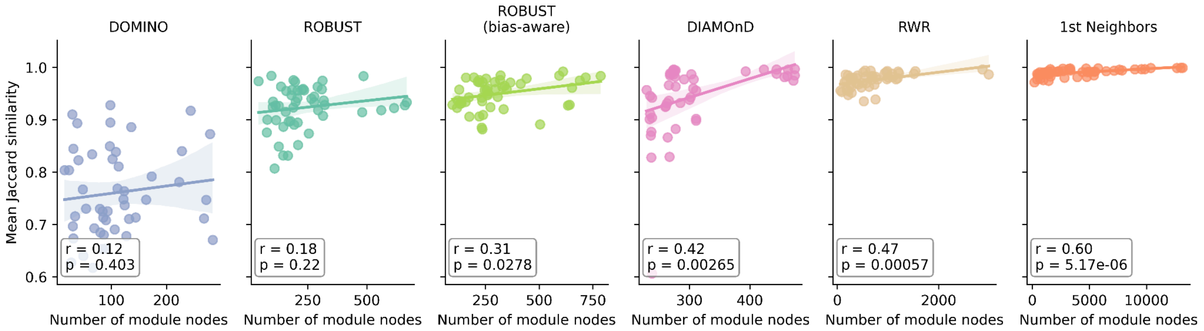

Figure S6: *Correlations between robustness and module size (expressed through the number of included nodes) for different AMIMs. r indicates the Pearson correlation and p the corresponding p-value.*

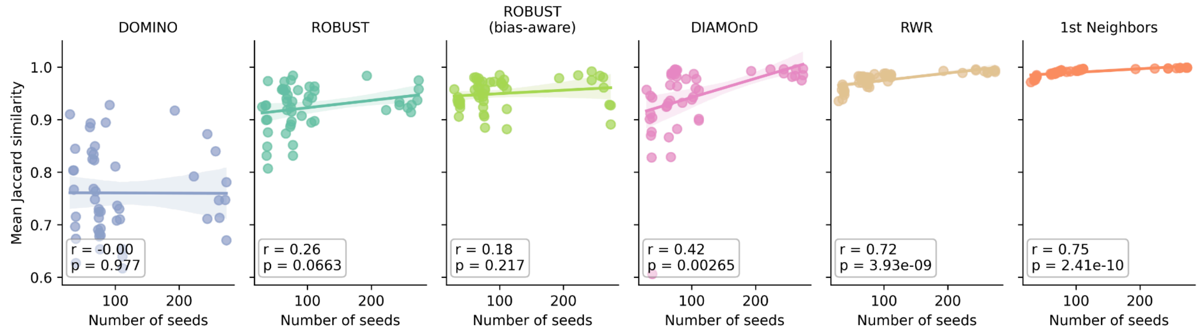

Figure S7: *Correlations between robustness to leave-one-out perturbations and the number of input seeds for different AMIMs.* r *indicates the Pearson correlation and* p *the corresponding p-value.*

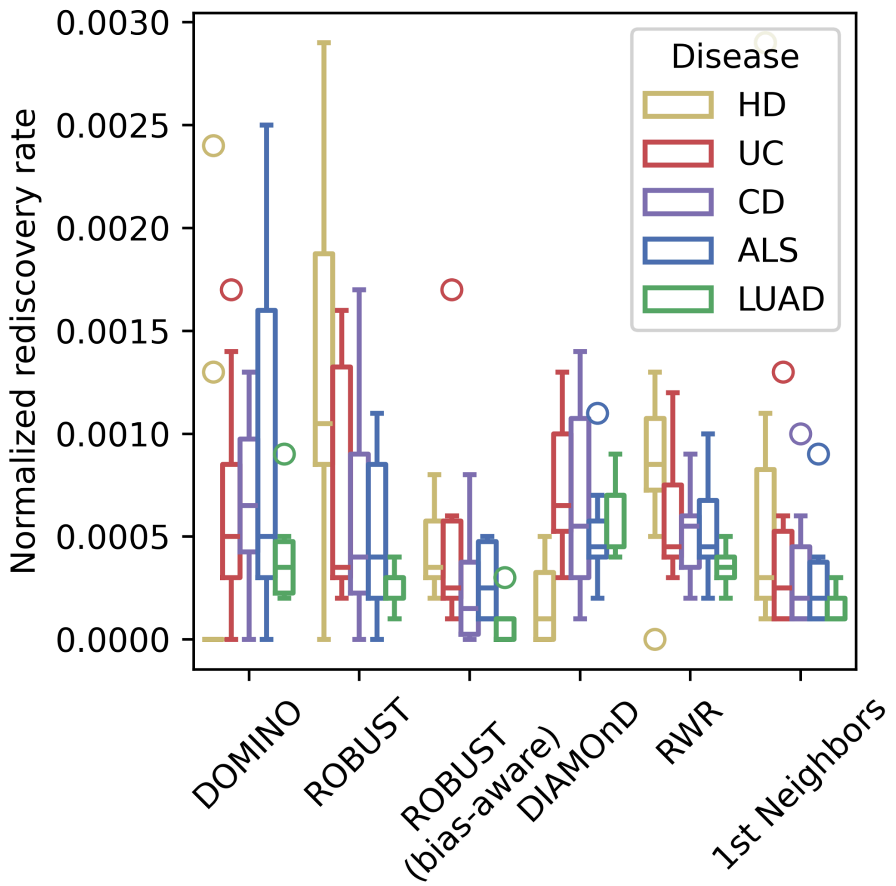

Figure S8: *Results of the seed rediscovery analysis for different diseases. Boxplots summarize results aggregated across all input networks.*

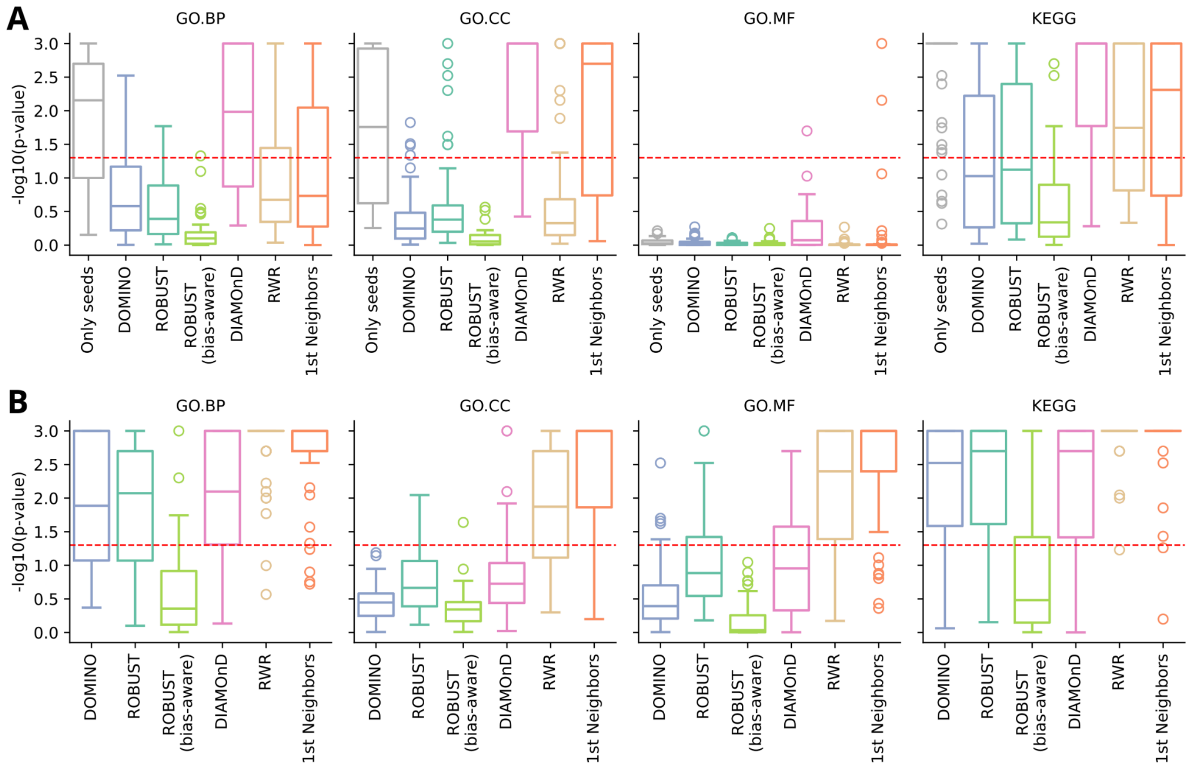

Figure S9: *Results of the functional coherence analysis with DIGEST in (**A**) reference-free mode and (**B**) reference-based mode. Functional coherence is expressed through empirical p-values transformed using* $-\,log_{10}$*. The significance level of 0.05 is indicated by the dashed line. A single boxplot summarizes the results for one AMIM in combination with one gene set source (GO.BP, GO.CC, GO.MF, or KEGG) aggregated across different seed set–network combinations.*

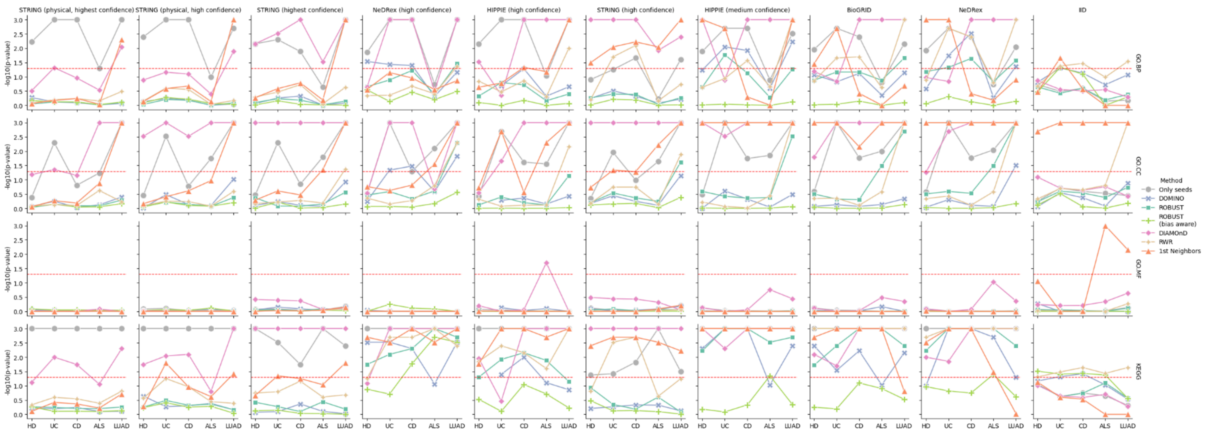

Figure S10: *Results of the functional coherence analysis with DIGEST in reference-free mode for different AMIM, network, gene set source (GO.BP, GO.CC, GO.MF, or KEGG), and disease combinations. Functional coherence is expressed through empirical p-values transformed using* $-\log_{10}$*. The significance level of 0.05 is indicated by the dashed line.*

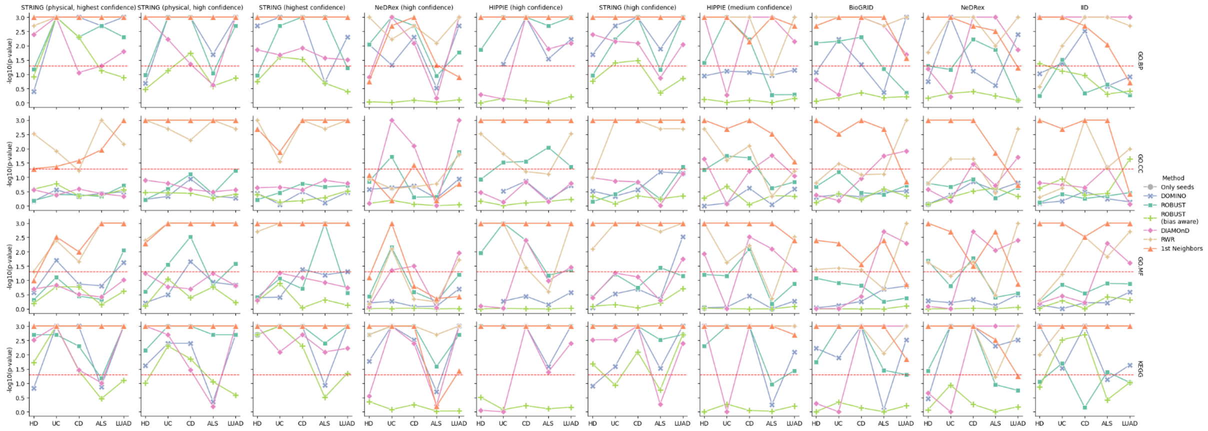

Figure S11: *Results of the functional coherence analysis with DIGEST in reference-based mode for different AMIM, network, gene set source (GO.BP, GO.CC, GO.MF, or KEGG), and disease combinations. Functional coherence is expressed through empirical p-values transformed using* $-\log_{10}$. *The significance level of 0.05 is indicated by the dashed line.*

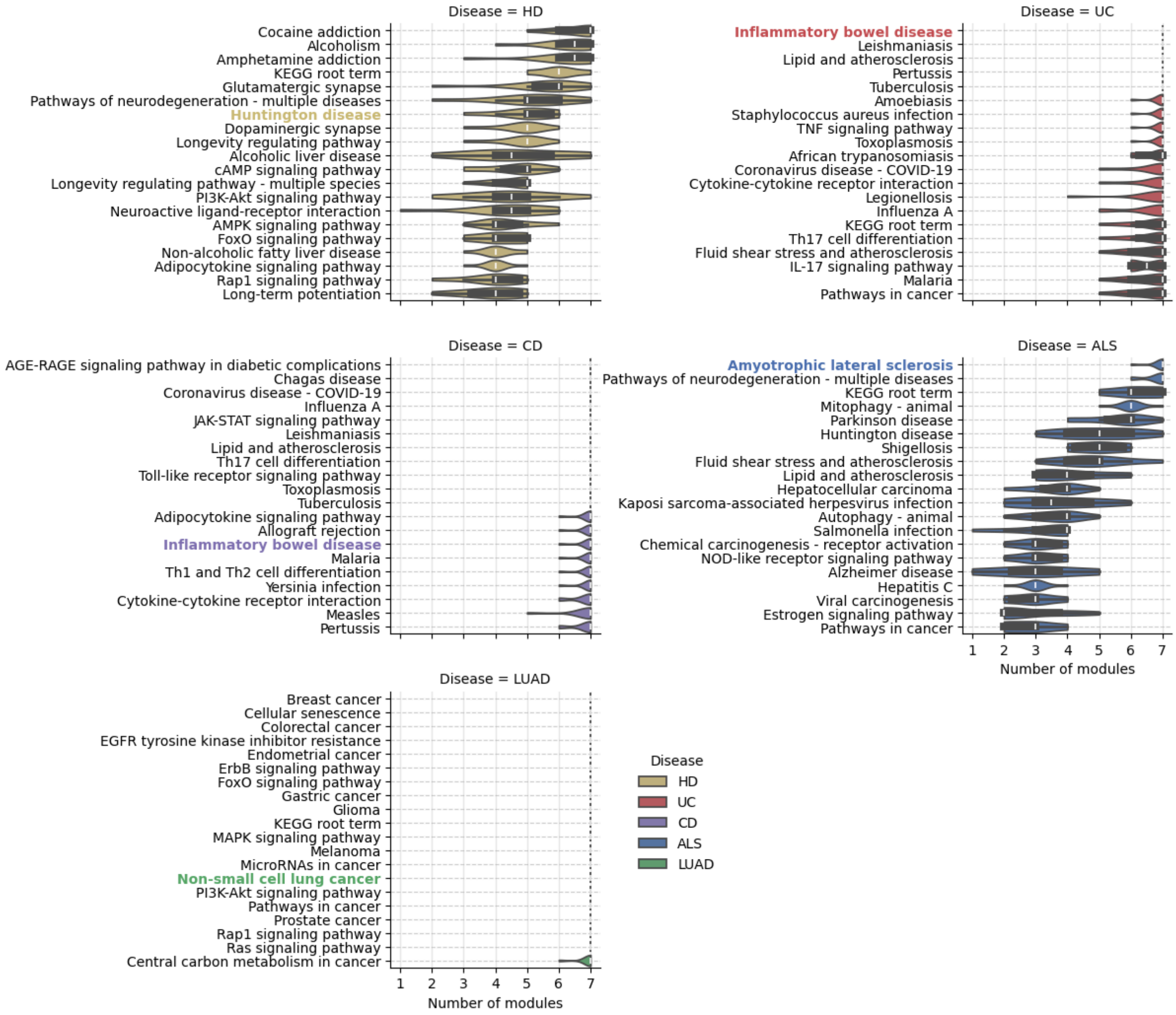

Figure S12: *Frequently enriched KEGG pathways. For each disease, the 20 most frequently enriched pathways are shown together with the number of modules in which they are enriched. The maximum possible number of modules per pathway is seven (six AMIMs plus the seed set). Pathways were considered enriched if they reached a corrected p-value < 0.05. A single violin summarizes, for each disease–pathway pair, the frequency of enrichment aggregated across all networks. Pathways are ranked by their enrichment frequency across all modules inferred for the respective disease (across all AMIMs and networks). The KEGG disease pathway most closely corresponding to the disease under study is highlighted in color (HD: Huntington disease; UC and CD: Inflammatory bowel disease; ALS: Amyotrophic lateral sclerosis; LUAD: Non-small cell lung cancer).*

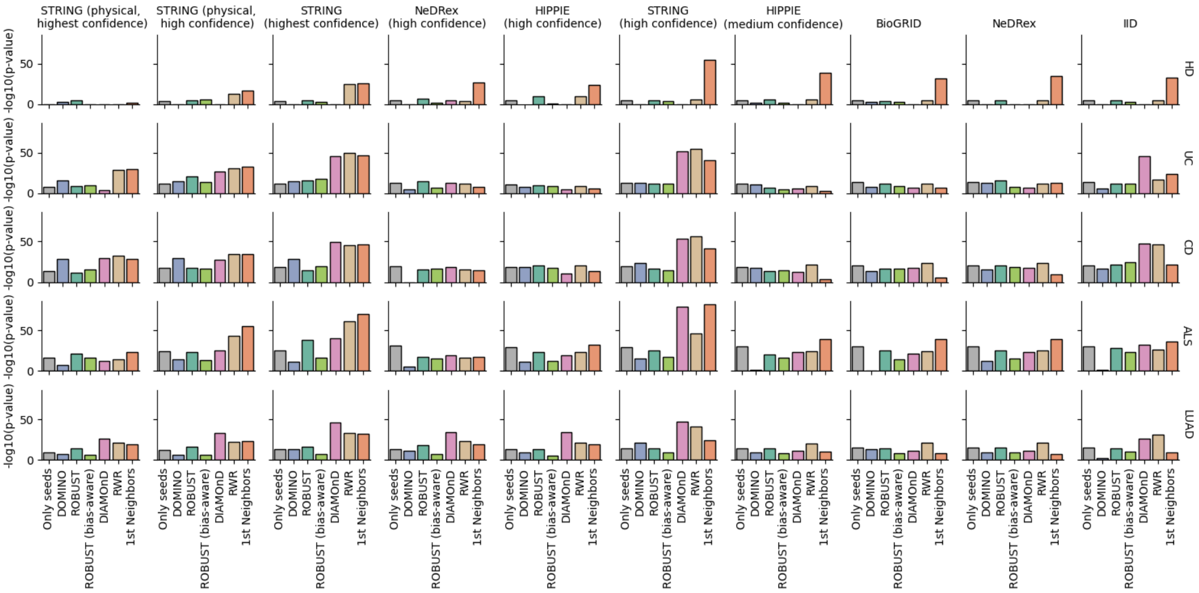

Figure S13: *Enrichment of KEGG disease pathways for modules inferred using different networks (columns), diseases (rows), and AMIMs. For each disease, the* $-\log_{10}$*-transformed corrected enrichment p-values for the KEGG disease pathway most closely matching the disease under study are shown (HD: Huntington disease; UC and CD: Inflammatory bowel disease; ALS: Amyotrophic lateral sclerosis; LUAD: Non-small cell lung cancer).*

References

1. Gene Ontology Consortium, Aleksander SA, Balhoff J, Carbon S, Cherry JM, Drabkin HJ, et al. The Gene Ontology knowledgebase in 2023. Genetics. 2023;224: iyad031. doi:10.1093/genetics/iyad031

2. Ashburner M, Ball CA, Blake JA, Botstein D, Butler H, Cherry JM, et al. Gene ontology: tool for the unification of biology. The Gene Ontology Consortium. Nat Genet. 2000;25: 25–29. doi:10.1038/75556

3. Kanehisa M, Furumichi M, Sato Y, Matsuura Y, Ishiguro-Watanabe M. KEGG: biological systems database as a model of the real world. Nucleic Acids Res. 2025;53: D672–D677. doi:10.1093/nar/gkae909

4. Ghiassian SD, Menche J, Barabási A-L. A DIseAse MOdule Detection (DIAMOnD) algorithm derived from a systematic analysis of connectivity patterns of disease proteins in the human interactome. PLoS Comput Biol. 2015;11: e1004120. doi:10.1371/journal.pcbi.1004120

5. Levi H, Elkon R, Shamir R. DOMINO: a network-based active module identification algorithm with reduced rate of false calls. Mol Syst Biol. 2021;17: e9593. doi:10.15252/msb.20209593

6. Bernett J, Krupke D, Sadegh S, Baumbach J, Fekete SP, Kacprowski T, et al. Robust disease module mining via enumeration of diverse prize-collecting Steiner trees. Bioinformatics. 2022;38: 1600–1606. doi:10.1093/bioinformatics/btab876

7. Kolberg L, Raudvere U, Kuzmin I, Vilo J, Peterson H. gprofiler2 -- an R package for gene list functional enrichment analysis and namespace conversion toolset g:Profiler. F1000Res. 2020;9: 709. doi:10.12688/f1000research.24956.2

8. Szklarczyk D, Nastou K, Koutrouli M, Kirsch R, Mehryary F, Hachilif R, et al. The STRING database in 2025: protein networks with directionality of regulation. Nucleic Acids Res. 2025;53: D730–D737. doi:10.1093/nar/gkae1113

9. Di Tommaso P, Chatzou M, Floden EW, Barja PP, Palumbo E, Notredame C. Nextflow enables reproducible computational workflows. Nat Biotechnol. 2017;35: 316–319. doi:10.1038/nbt.3820

10. Kurtzer GM, Sochat V, Bauer MW. Singularity: Scientific containers for mobility of compute. PLoS One. 2017;12: e0177459. doi:10.1371/journal.pone.0177459

11. Ewels PA, Peltzer A, Fillinger S, Patel H, Alneberg J, Wilm A, et al. The nf-core framework for community-curated bioinformatics pipelines. Nat Biotechnol. 2020;38: 276–278. doi:10.1038/s41587-020-0439-x

12. Peixoto TP. The graph-tool python library. figshare; 2017. doi:10.6084/M9.FIGSHARE.1164194.V14

13. Tweedie S, Braschi B, Gray K, Jones TEM, Seal RL, Yates B, et al. Genenames.Org: The HGNC and VGNC resources in 2021. Nucleic Acids Res. 2021;49: D939–D946. doi:10.1093/nar/gkaa980

14. Maglott D, Ostell J, Pruitt KD, Tatusova T. Entrez Gene: gene-centered information at NCBI. Nucleic Acids Res. 2007;35: D26–31. doi:10.1093/nar/gkl993

15. Dyer SC, Austine-Orimoloye O, Azov AG, Barba M, Barnes I, Barrera-Enriquez VP, et al. Ensembl 2025. Nucleic Acids Res. 2025;53: D948–D957. doi:10.1093/nar/gkae1071

16. UniProt Consortium. UniProt: The universal protein knowledgebase in 2025. Nucleic Acids Res. 2025;53: D609–D617. doi:10.1093/nar/gkae1010

17. Szklarczyk D, Kirsch R, Koutrouli M, Nastou K, Mehryary F, Hachilif R, et al. The STRING database in 2023: protein-protein association networks and functional enrichment analyses for any sequenced genome of interest. Nucleic Acids Res. 2023;51: D638–D646. doi:10.1093/nar/gkac1000

18. Oughtred R, Rust J, Chang C, Breitkreutz B-J, Stark C, Willems A, et al. The BioGRID database: A comprehensive biomedical resource of curated protein, genetic, and chemical interactions. Protein Sci. 2021;30: 187–200. doi:10.1002/pro.3978

19. Alanis-Lobato G, Andrade-Navarro MA, Schaefer MH. HIPPIE v2.0: enhancing meaningfulness and reliability of protein-protein interaction networks. Nucleic Acids Res. 2017;45: D408–D414. doi:10.1093/nar/gkw985

20. Kotlyar M, Pastrello C, Ahmed Z, Chee J, Varyova Z, Jurisica I. IID 2021: towards context-specific protein interaction analyses by increased coverage, enhanced annotation and enrichment analysis. Nucleic Acids Res. 2022;50: D640–D647. doi:10.1093/nar/gkab1034

21. Sadegh S, Skelton J, Anastasi E, Bernett J, Blumenthal DB, Galindez G, et al. Network medicine for disease module identification and drug repurposing with the NeDRex platform. Nat Commun. 2021;12: 6848. doi:10.1038/s41467-021-27138-2

22. Aguirre-Plans J, Gallego X, Guney E. Optimizing protein-protein interaction filtering based on detection methods to construct high-quality interactomes for network medicine. Optimizing Protein-Protein Interaction Filtering Based on Detection Methods to Construct High-Quality Interactomes for Network Medicine. REPO4EU; 2025. doi:10.58647/rexpo.25000080.v1

23. Blondel VD, Guillaume J-L, Lambiotte R, Lefebvre E. Fast unfolding of communities in large networks. J Stat Mech. 2008;2008: P10008. doi:10.1088/1742-5468/2008/10/P10008

24. Johnson DS, Minkoff M, Phillips SJ. The prize collecting Steiner tree problem: theory and practice. Symposium on Discrete Algorithms. 2000;1: 760–769. Available: https://citeseerx.ist.psu.edu/document?repid=rep1&type=pdf&doi=7090d6e01846f2a8b0759b370bf5b0e14dd6fd90

25. Sarkar S, Lucchetta M, Maier A, Abdrabbou MM, Baumbach J, List M, et al. Online bias-aware disease module mining with ROBUST-Web. Bioinformatics. 2023;39: btad345. doi:10.1093/bioinformatics/btad345

26. Schaefer MH, Serrano L, Andrade-Navarro MA. Correcting for the study bias associated with protein-protein interaction measurements reveals differences between protein degree distributions from different cancer types. Front Genet. 2015;6: 260. doi:10.3389/fgene.2015.00260

27. Köhler S, Bauer S, Horn D, Robinson PN. Walking the interactome for prioritization of candidate disease genes. Am J Hum Genet. 2008;82: 949–958. doi:10.1016/j.ajhg.2008.02.013

28. Cowen L, Ideker T, Raphael BJ, Sharan R. Network propagation: a universal amplifier of genetic associations. Nat Rev Genet. 2017;18: 551–562. doi:10.1038/nrg.2017.38

29. Elbatreek MH, Sadegh S, Anastasi E, Guney E, Nogales C, Kacprowski T, et al. NOX5-induced uncoupling of endothelial NO synthase is a causal mechanism and theragnostic target of an age-related hypertension endotype. PLoS Biol. 2020;18: e3000885. doi:10.1371/journal.pbio.3000885

30. Agrawal A, Balcı H, Hanspers K, Coort SL, Martens M, Slenter DN, et al. WikiPathways 2024: next generation pathway database. Nucleic Acids Res. 2024;52: D679–D689. doi:10.1093/nar/gkad960

31. Milacic M, Beavers D, Conley P, Gong C, Gillespie M, Griss J, et al. The Reactome Pathway Knowledgebase 2024. Nucleic Acids Res. 2024;52: D672–D678. doi:10.1093/nar/gkad1025

32. Reimand J, Kull M, Peterson H, Hansen J, Vilo J. g:Profiler--a web-based toolset for functional profiling of gene lists from large-scale experiments. Nucleic Acids Res. 2007;35: W193–200. doi:10.1093/nar/gkm226

33. Adamowicz K, Maier A, Baumbach J, Blumenthal DB. Online in silico validation of disease and gene sets, clusterings or subnetworks with DIGEST. Brief Bioinform. 2022;23. doi:10.1093/bib/bbac247

34. Demir E, Cary MP, Paley S, Fukuda K, Lemer C, Vastrik I, et al. The BioPAX community standard for pathway data sharing. Nat Biotechnol. 2010;28: 935–942. doi:10.1038/nbt.1666

35. Gyori BM, Hoyt CT. PyBioPAX: biological pathway exchange in Python. J Open Source Softw. 2022;7: 4136. doi:10.21105/joss.04136

36. Maier A, Hartung M, Abovsky M, Adamowicz K, Bader GD, Baier S, et al. Drugst.One - a plug-and-play solution for online systems medicine and network-based drug repurposing. Nucleic Acids Res. 2024. doi:10.1093/nar/gkae388

37. Gyöngyi Z, Garcia-Molina H, Pedersen J. Combating Web Spam with TrustRank. Proceedings 2004 VLDB Conference. Elsevier; 2004. pp. 576–587. doi:10.1016/b978-012088469-8.50052-8

38. Ewels P, Magnusson M, Lundin S, Käller M. MultiQC: summarize analysis results for multiple tools and samples in a single report. Bioinformatics. 2016;32: 3047–3048. doi:10.1093/bioinformatics/btw354
